# Contrasting Association Between COVID-19 Vaccine Hesitancy and Mental Health Status in India and Saudi Arabia—A Preliminary Evidence Collected During the Second Wave of COVID-19 Pandemic

**DOI:** 10.3389/fmed.2022.900026

**Published:** 2022-05-04

**Authors:** Saikarthik Jayakumar, Saraswathi Ilango, Senthil Kumar K., Abdullah Alassaf, Abdullah Aljabr, Anand Paramasivam, Suresh Mickeymaray, Yazeed Mohammed Hawsah, Ahmed Saad Aldawish

**Affiliations:** ^1^Department of Basic Medical Sciences, College of Dentistry, Majmaah University, Al Majma’ah, Saudi Arabia; ^2^Department of Medical Education, College of Dentistry, Majmaah University, Al Majma’ah, Saudi Arabia; ^3^Department of Physiology, Madha Medical College and Research Institute, Chennai, India; ^4^Department of Preventive Dental Science, College of Dentistry, Majmaah University, Al Majma’ah, Saudi Arabia; ^5^Department of Biology, College of Science, Majmaah University, Al Majma’ah, Saudi Arabia; ^6^College of Dentistry, Majmaah University, Al Majma’ah, Saudi Arabia

**Keywords:** vaccine hesitancy, COVID-19 vaccine hesitancy scale, COVID-19 vaccines, mental health, depression, anxiety, PTSD, mental health support

## Abstract

**Background:**

Vaccine hesitancy is a global public health threat. Understanding the role of psychological factors in vaccine hesitancy is often neglected and relatively less explored.

**Aim and Objectives:**

To analyze the relationship between mental health and COVID-19 vaccine hesitancy before and after the advent of COVID-19 vaccines (AC19V) in the general population of India and Saudi Arabia (KSA) which vary in severity of the pandemic and vaccine mandates.

**Materials and Methods:**

A total of 677 adult participants from India and KSA participated in this cross-sectional online web-based survey. Sociodemographic details and current COVID-19 status pertaining to infection and vaccination were collected. Depression, anxiety, post-traumatic stress disorder (PTSD) symptoms, and perceptive need for mental health support (MHS) were assessed before and after AC19V. A newly constructed and validated COVID19 vaccine hesitancy scale-12 (COVID19-VHS12) scale was used to evaluate the COVID-19 vaccine hesitancy.

**Results:**

Prevalence and levels of depression and anxiety symptoms decreased significantly in Saudis but not in Indians after AC19V. PTSD symptoms showed a significant reduction in both India and KSA. Anxiety symptoms were higher in KSA than India before AC19V while PTSD was higher in India before and after AC19V. Except for the place of residence and employment status, the subgroups of sociodemographic variables which were at higher risk of negative mental health before AC19V showed improvement in their mental health after AC19V. The prevalence of COVID-19 vaccine hesitancy in India and KSA was 50.8% (95% CI 45.73–55.89%) and 55.7% (95% CI 50.16–61.31%), respectively. A bidirectional association between vaccine hesitancy and mental health was observed in KSA but not in India. Higher vaccine hesitancy favored higher levels of depression, anxiety, and perceptive need for MHS and vice versa in KSA. None of the mental health parameters predicted vaccine hesitancy in India, while higher vaccine hesitancy increased the risk of anxiety.

**Conclusion:**

Vaccine hesitancy has a negative impact on mental health and vice versa over and above the impact of sociodemographic factors and COVID-19 vaccination and infection status which shows variations between India and KSA.

## Introduction

As of 1st December 2020, globally, there were 61.8 million reported cases of COVID-19 and 1.4 million deaths since the start of the pandemic (1). On 2nd December 2020, the United Kingdom’s Medicine and Healthcare products regulatory agency (MHPRA) approved the world’s first vaccine against COVID-19, Pfizer-BioN Tech Vaccine, on a temporary emergency basis (2). The center for disease control and prevention (CDC) has stated that the number of new cases and deaths due to COVID-19 was much lower among vaccinated population, especially among the elder population (3). The World health organization (WHO) has also urged people across the globe to get vaccinated, although cautioning that the vaccine is not 100% effective (4).

Despite the benefits of vaccines, WHO has warned against vaccine mandates unless all the options available are exhausted. However, with the spread of the highly contagious delta variant of SARS-CoV-2, some countries executed stringent measures to improve the vaccine rate in their population. Countries like Austria, France, Germany, Italy, Morocco, Canada, United States, and United Kingdom have declared COVID-19 vaccines mandates ranging from permission for allowing access to malls, bars, public, and private establishments to mandating for selected sectors of the population (5). Saudi Arabia has rigid COVID-19 vaccine rules. On 18th May 2021, the Ministry of Interior (MOI) of Saudi Arabia announced vaccine mandates starting from 1st August 2020, for entering all governmental and private educational facilities, establishments, entertainment and sporting events, and public transportation (6). These mandates have resulted in a rise in vaccine rates, a fall in COVID-19 cases, and a rise in workplace visits based on Google mobility data (7). On the other hand, many countries are not keen on vaccine mandates. In India, the ministry of family welfare and health had explicitly stated that getting vaccinated against COVID-19 is voluntary (8). While there have been reports of coercive vaccination by local authorities and employers, the principal reasons behind the delay in achieving desired vaccine rates are vaccine hesitancy and lack of availability and access to vaccines (9). The severity of the pandemic varies between India and Saudi Arabia. Currently, India is the second worst hit country due to COVID-19 only behind the United States. As of 27th February 2022, the total number of SARS-CoV-2 infected cases was 42.9 million, with 514,000 deaths (10). At the same date, Saudi Arabia has reported 744,000 positive cases and 8,994 deaths (10). At the time of manuscript preparation, about 72.3% and 61.1% of the Indian population and 75.7% and 70.9% of the total Saudi population have taken the first dose and second doses of COVID-19 vaccine, respectively (11).

Regardless of the evidence of improved public health to infectious disease, vaccine hesitancy has been a significant area of attention and concern (12). In 2019, the WHO stated that vaccine hesitancy is one of the top 10 global threats to public health (13). It is the tendency of delay in acceptance or refusal to get vaccinated despite the availability of vaccines. As vaccine hesitancy involves many factors, addressing them is not an easy task. Geography, culture, socioeconomic status (14), and behavioral factors such as complacency, confidence, and convenience (15) have been linked to vaccine hesitancy. Globally only a handful of countries have been reported to have no vaccine hesitancy (7/194) (16). Irrespective of economic status, vaccine hesitancy has been noted in mass vaccination campaigns across low-, middle-, and high-income countries (17–19). Recent studies have shown that COVID-19 vaccine hesitancy is highest among the Middle East and North African countries, Europe and Central Asia, Western and Central Africa (20). Among African countries, Cameroon, Senegal, and Liberia had the highest vaccine hesitancy due to lower trust in manufacturing companies (21, 22). Studies in Asia pacific region revealed that Hong Kong, Japan, and Taiwan had higher rates of hesitancy to get vaccinated against COVID-19 (23–25). Mistrust on healthcare providers was the reason behind high vaccine hesitancy amongst Western Europe and Central Asia (26). Urrunaga-Pastor et al. studies have shown that the vaccine acceptance rates were higher in Latin American and Caribbean countries (27). With the exception of Israel and the United Arab Emirates, vaccine hesitancy is very high among the MENA countries (28). Geospatial disparity and low trust were common reasons for higher vaccine hesitancy in the United States and Central Europe (29–31).

Vaccine hesitancy has many reasons, the most common reason is risk-benefit evidence (less than 25%). This was linked with safety concerns and fear of side effects due to the vaccine (32). Understanding the role of psychological factors in vaccine hesitancy is often underplayed and needs to be explored (33). Globally, very few studies have identified the effect of mental health on vaccine hesitancy. There has been evidence of inconsistent results about the association between mental health status and willingness to get vaccinated. Earlier studies assessing vaccine hesitancy have demonstrated that poor mental health is associated with higher vaccine acceptance toward influenza vaccines (34, 35). In studies conducted assessing the relation of mental health with COVID-19 vaccine hesitancy there were conflicting results across countries (36–38). At the time of the manuscript preparation (18th April, 2022), to our knowledge there were no studies that evaluated the impact of mental health on vaccine hesitancy in India and Saudi Arabia population. Both these countries have been reported to have poor sleep quality and psychological distress during the COVID-19 pandemic (39, 40). Such vulnerable subgroups needed to be prioritized in getting vaccinated (41) just like those with co-morbidities like diabetes mellitus and hypertension.

We hypothesized that with the advent of COVID-19 vaccines, the psychological distress experienced by the public would be eased. However, including the impact of vaccine hesitancy on mental health and vice versa called for ambiguity. Hence, we decided to study the mutual impact of vaccine hesitancy on mental health, if any, in relation to the advent of COVID-19 vaccines. To our knowledge, this is the first study to analyze the relationship between mental health and vaccine hesitancy before and after the advent of COVID-19 vaccines in the general population of India and Saudi Arabia. We also intend to compare the effect of various factors affecting vaccine hesitancy and the influence of mental health. In addition, we also decided to compare the results between India and Saudi Arabia, two countries that are both Asian countries but vary in terms of ethnicity, culture, religion, government type, per capita income, healthcare in addition to the severity of the pandemic and vaccine mandates.

## Materials and Methods

### Study Design

**FIGURE 1:**
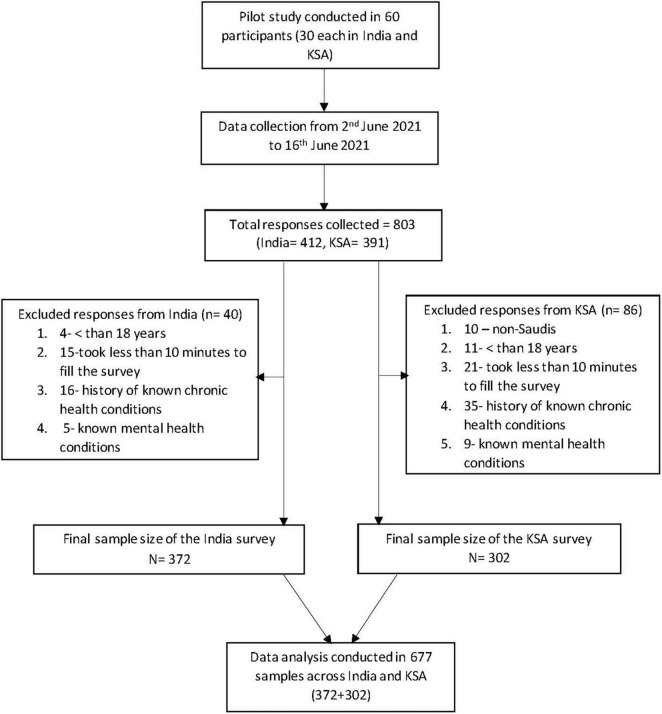
Flow chart illustrating the sample selection in India and KSA. KSA, Kingdom of Saudi Arabia.

The study was conducted using a cross-sectional design in India and Saudi Arabia. The study was conducted following STROBE guidelines for cross-sectional study (Figure 1) (42). Data were collected from 2nd to 16th June 2021, using Computer Assisted Web Interviews by snowball sampling technique. The Google form link was distributed through WhatsApp to all potential participants, and the link was also posted on the Facebook wall, the WhatsApp status, and the twitter handle of all authors in this study. Only those above 18 years and citizens of India and Saudi Arabia residing in their respective countries were asked to participate in the survey. Those who spent lesser than 10 min to fill the survey forms, those with a history of mental health disorders and chronic diseases were excluded from the study.

### Sample Size

Employing the method by R Hill (43), as a rule of thumb, the minimal sample size should be at least ten times higher than the number of variables in the study (43). The present study has a total of 18 variables, and so the minimal sample size that would be needed for the study is around 180. We calculated the minimum sample size required for the study using an online sample size calculation tool.^1^ With precision at 5%, level of confidence at 95%, and considering the prevalence of COVID-19 vaccine hesitancy in India and Saudi Arabia to be 23% (44) and 24.55% (45), respectively, the minimum sample size was calculated to be 285 and 273, respectively. Considering a non-response rate of 10%, the final sample size needed was 313 and 300 for India and Saudi Arabia, respectively. We collected 412 responses from India and 391 responses from Saudi Arabia. 40 responses from India and 86 responses from Saudi Arabia were not eligible as they did not meet the inclusion criteria. The final working sample size of the study was 372 in India and 305 in Saudi Arabia (Figure 1).

### Survey Instrument

The survey questionnaire included six sections. The first section described the purpose of the study, ethical approval details, willingness to participate, data anonymity and data confidentiality. Once the participants agreed, the questionnaire moved to section “II.” All the questions in the survey were mandatory to be filled. However, the participants were free to exit the survey as and when they pleased. No incentives or rewards in any form were offered for participation. Section “II” collected sociodemographic details such as age, gender, educational qualification, monthly income, place of residence (urban/rural), marital status, occupational status (healthcare/non-healthcare professional). Any known history of chronic health conditions and mental health disorders were also collected.

Section “III” included details pertaining to current COVID-19 status regarding infection and vaccination. The items were whether tested positive for COVID-19 (Yes/No), the present status of COVID-19 vaccination (Yes-1st dose/Yes-2nd dose/No), whether the participants think COVID-19 vaccine is beneficial (Yes/No), whether the participants developed active COVID-19 after vaccination (Yes/No/I don’t know).

Section “IV” comprised of questions related to the hesitancy of the participants toward getting vaccinated for COVID-19, which was collected using a self-administered COVID-19 vaccine hesitancy scale made of 12 items (described below). Sections “V” and “VI” assessed the mental health status of the participants using the screening tools, Patient health questionnaire-2 (PHQ-2), Generalized Anxiety Disorder-2 item (GAD-2), Impact of Event Scale-6 (IES-6), and a single item for the perceptive need for mental health support (MHS). In section “V,” the participants were asked, “before December 2020, how often were you bothered by the following problems.” In section “VI” the participants were asked “When filling this survey, how often in the last 2 weeks, were you bothered by the following problems.” Sections “V” and “VI” screened for the symptoms of depression, anxiety, PTSD, and perceptive need for MHS before and after the advent of the COVID-19 vaccine (AC19V), respectively.

The questionnaire used in India were deployed in English. For the study in Saudi Arabia, all the questions were translated into Arabic. The translated version was again retranslated to English to check for clarity of the questions. This translation-retranslation was done by a native Arabic speaker proficient in both English and Arabic (46). A pilot study was conducted prior to the primary survey, in 60 participants with 30 each in India and Saudi Arabia, to check for face validity and average duration to fill the questionnaire. Feedback was collected from the participants, and necessary modifications in the form of simplification of phrasing and vocabulary were made to improve the clarity and simplicity of the questionnaire.

#### Development of COVID-19 Vaccine Hesitancy Scale-12 Items and Psychometric Analysis

The COVID-19 vaccine hesitancy scale used in the present study was adopted from multiple studies conducted earlier (47–51). The items included were created after extensive literature review, discussion with local experts and peers. Since negative information, personal and family circumstances, and fear can contribute to decision making, negative items were added to the scale (52). Such items were scored on a Likert scale ranging from 1- highly disagree to 5- highly agree. Positive items were constructed and were reverse coded to measure confidence and trust on vaccines. A mix of both positive and negative questions removes response bias from the participants and improves the reliability of the results obtained (53).

#### Identification of Latent Variables Using Exploratory Factor Analysis

**FIGURE 2:**
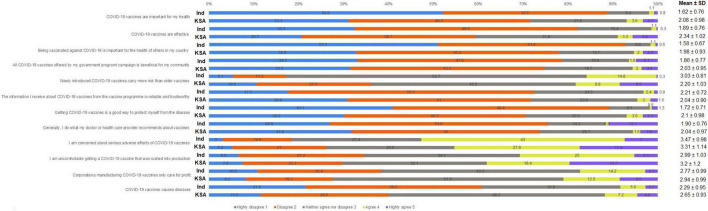
Distribution of responses for each of the 12 items of COVID-19 vaccine hesitancy Scale (in %) with Mean and Standard deviation score of each item in India and Saudi Arabia. Questions 5, 9, 10, 11 and 12 were coded with 1–highly disagree to 5–highly agree. Questions 1, 2, 3, 4, 6, 7 and 8 were reverse coded with 1–highly agree and 5–highly disagree, Ind, India; KSA, Kingdom of Saudi Arabia.

The 12 items of the COVID-19 vaccine hesitancy scale-12 (COVID19-VHS12) scale were analyzed using exploratory factor analysis (EFA) to identify the latent variables using principal component analysis with varimax rotation. The extracted factors were analyzed for retention using scree plot and Kaiser criterion with Eigen value > 1 and counter validated using parallel analysis. We obtained two factors named Negative and Positive attitude toward the COVID-19 vaccine. Question 5, 9, 10, 11, and 12 were included in the factor-negative attitude toward COVID-19 vaccines, and the remaining questions 1, 2, 3, 4, 6, 7, and 8 were included in the factor—positive attitude toward COVID-19 vaccine. The score of the COVID19-VHS12 was calculated by the summation of individual scores of the 12 items (maximum score 60). The items of the COVID19-VHS12 scale are given in Figure 2.

#### Confirmatory Factor Analysis and Reliability Analysis

The extracted items under the two factors were further analyzed for model fit using confirmatory factor analysis (CFA). Standardized regression weights of < 0.6 were considered as poor loadings. The goodness of fit for the COVID19-VHS12 with two factors had the following indices. For the English version of the scale, root mean square error of approximation (RMSEA) = 0.064; comparative fit index (CFI) = 0.936; Tucker-Lewis index (TLI) = 0.920. For the Arabic version, RMSEA = 0.077; CFI = 0.96; TLI = 0.95. The two-factor solution obtained from EFA demonstrated a good model fit for both the English and Arabic version of the COVID19-VHS12 based on the above-mentioned goodness of fit indices (54).

Further, reliability analysis was performed using Cronbach’s alpha coefficient. The Cronbach’s value for the positive and negative factors of COVID19-VHS12 for English version was 0.86 and 0.68 and for Arabic version was 0.94 and 0.79, respectively. Based on Cronbach’s value, the reliability of the two factors of COVID19-VHS12 ranged from acceptable to excellent for English and Arabic versions (55).

#### Determination of Cut Off Score for COVID19-Vaccine Hesitancy Scale-12

The Receiver operating characteristic (ROC) curve has been used previously to determine the cut-off scores of various scales (56, 57). The 12 items of COVID19-VHS12 were loaded as the test variable, and a single item binary variable of “Do you think COVID-19 Vaccine is beneficial (Yes/No)” was loaded as the state variable. The AUROC (Area Under Receiver Operating Characteristic Curve) value for the English version was 81.6 and for the Arabic version was 85.5. The cut-off value for English version was 27.5 (sensitivity 81.9% and specificity 36.7%). Arabic version also had the same cut-off score with the sensitivity of 86% and specificity of 30%. This was rounded off to 28, and any value above 28 was considered vaccine hesitant and scores ≤ 28 were considered not vaccine hesitant. The results of EFA, CFA, and ROC analysis are given in Supplementary Tables 16, 17 and Supplementary Figures 1–4.

#### Reliability Analysis for Mental Health Measures

PHQ-2, GAD-2, and IES-6 are brief screening tools to assess depression, anxiety, and PTSD symptoms, respectively. Earlier studies have used these tools in both countries (58–61). The Cronbach’s alpha score for PHQ-2, GAD-2, and IES-6 before AC19V was 0.45, 0.80, and 0.84 for Indian samples and 0.74, 0.82, and 0.80 for Saudi samples. Cronbach’s alpha score for PHQ-2, GAD-2, and IES-6 after AC19V was 0.70, 0.85, and 0.90 for Indian samples and 0.83, 0.86, and 0.86 for Saudi samples. All three scales demonstrated good internal consistency and test-retest reliability.

### Ethical Considerations

The study was approved by the Majmaah University Research Ethics Committee (MUREC-May.31/COM-2021/35-2) and Institutional Ethical Committee of Madha Medical College and Research Institute (No/009/2021/IEC/APP/MMC&RI). The study was conducted in adherence to Helsinki Declaration for research on human participants.

### Statistical Analysis

Descriptive statistics were done for all the variables. Cross-sectional analysis between variables across different subgroups and between the two countries was performed using the Mann-Whitney *U*-test, Kruskal Wallis test (continuous variables), and Chi-square test (categorical variables). Comparison of mental health parameters before and after AC19V was performed using Wilcoxon signed-rank test for continuous variables and McNemar’s test for categorical variables. Spearman’s correlation test was performed to study the correlation between all the obtained scores.

To study the association between mental health parameters and COVID-19 vaccine hesitancy, binary logistic regression analysis was used for depression, anxiety, and vaccine hesitancy, and generalized linear regression analysis was used for PTSD. To begin with, unadjusted bivariate regression analysis was performed with mental health parameters viz depression, anxiety, PTSD, and perceptive need for MHS before and after AC19V and vaccine hesitancy as the dependent variable and sociodemographic factors as the independent variable. Despite the results, we included all the sociodemographic variables, which are potential confounders in our adjusted regression models.

Three types of regression models were used to explore the contributory factors for each mental health parameter and COVID-19 vaccine hesitancy. Initially, unadjusted regression analysis (regression model 1) was performed, and the results were expressed as crude odds ratio (OR), 95% confidence interval (95% CI), and *P*-value. For COVID-19 vaccine hesitancy (dependent variable), COVID-19 related factors viz tested positive for COVID-19, COVID-19 vaccination status, active infection after COVID-19 vaccination and mental health parameters viz. depression, anxiety, PTSD, and perceptive need for MHS before and after AC19V were included as independent variables. For mental health status, depression, anxiety, PTSD, and perceptive need for MHS after AC19V were the dependent variables, and vaccine hesitancy, COVID-19 related factors, and the remaining mental health parameters were included as the independent variables.

In the second regression model, to study the impact of each independent variable over and above the influence of sociodemographic variables, each of the independent variable’s effect was adjusted for sociodemographic variables in separate regression models.

In the third regression model, to study the impact of each independent variable over and above the influence of sociodemographic variables and COVID-19 status in relation to infection and vaccination, the effect of each independent variable was adjusted for both sociodemographic variables and COVID-19 related factors in separate regression models. The results of the second and third regression models were expressed as adjusted odds ratio (aOR), 95% confidence interval (95% CI), and *P*-value.

Statistical analysis was performed using SPSS version 26 (IBM, NY, United States). Parallel analysis was performed using scripts from O’Connor (62). CFA was performed using SPSS AMOS version 23 (IBM, NY, United States). Statistical significance was set at two-tailed *P* < 0.05.

## Results

**FIGURE 3:**
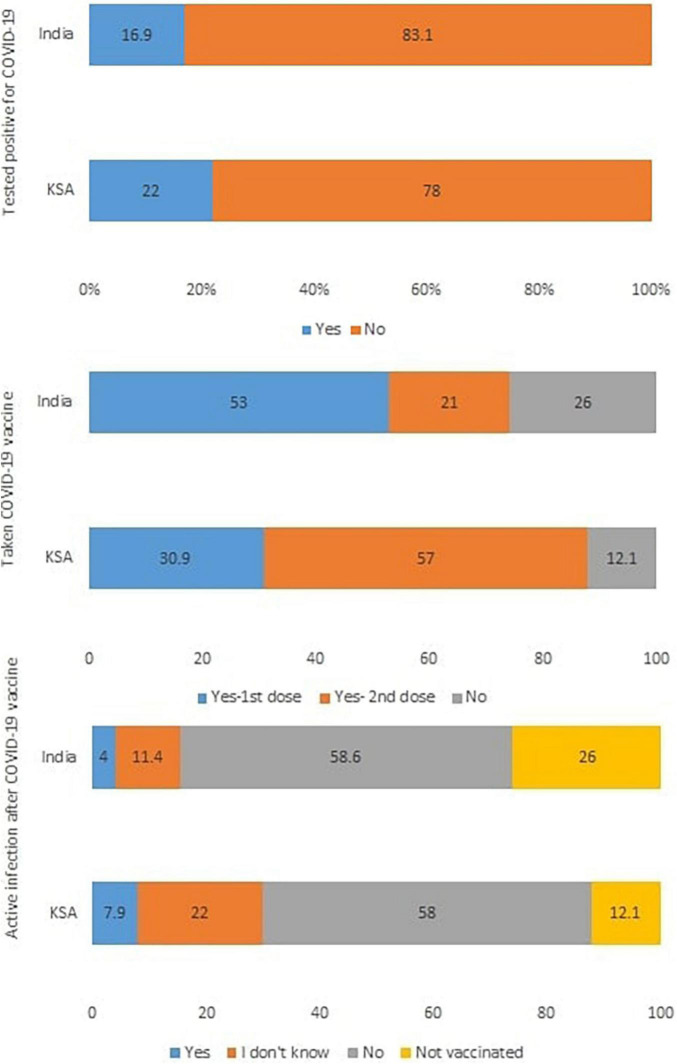
Distribution of responses to COVID-19 status in the self-administered questionnaire (in %). KSA, Kingdom of Saudi Arabia.

This bi-national survey includes 372 and 305 adult participants with an average age of 22.18 ± 6.87 (18–53) and 25.37 ± 9.29 (18–58) years from India and Saudi Arabia, respectively. In both the nations, majority of the participants were females (63.7%—India, 65.6%—KSA), unmarried i.e., single, divorced, or widowed (89.5%—India, 71.5%—KSA), with undergraduate level of education (90.3%—India, 71.4%—KSA), and living in urban areas (68%—India, 82.6%—KSA). The majority of the study participants from India were students in the healthcare field (59.1%) and without income (81.5%), while the majority of the Saudi participants were non-healthcare workers and unemployed individuals (78%) and those with monthly income below 10,000 SAR (54.1%). 16.9 and 22% of the participants had tested positive for COVID-19 in India and Saudi Arabia, respectively. More than twice the number of Indians (26%) were not vaccinated against COVID-19 when compared to that of Saudi Arabia (12.1%) (Figure 3).

### Comparison of Mental Health Parameters Before and After the Advent of COVID-19 Vaccines

There was a significant reduction in both scores (*P* = 0.001, 0.002) and prevalence (*P*-value = 0.002, 0.035) of depression and anxiety in the Saudi population after AC19V, while no significant changes were observed in India. PTSD scores showed significant reduction after AC19V in both India (*P* < 0.001) and Saudi Arabia (*P* = 0.017). Anxiety scores were significantly higher (*P* = 0.012) in Saudi Arabia than in India before AC19V. PTSD symptoms were significantly higher in India when compared to Saudi Arabia both before and after AC19V (*P* < 0.001) (Table 1).

**TABLE 1:** Comparison of mental health parameters before and after the advent of COVID-19 vaccines.

Country	Mental health parameter	Before	After	Negative rank (after < before)	Positive rank (after > before)	Ties (after = before)	*P*-value
India (*N* = 372)	Depression Mean ± *SD*	2.02 ± 1.664	2.04 ± 1.851	86	79	207	0.956^a^
	*N* (%)	134 (36%)	128 (34.4%)	39	33	300	0.556^b^
KSA (*N* = 305)	Depression Mean ± *SD*	2.28 ± 1.917	1.99 ± 1.972	90	51	164	**0.001^a^**
	*N* (%)	118 (38.7%)	93 (30.5%)	42	17	246	**0.002^b^**
*P*-value^c^	–	0.183	0.433	–	–	–	–
*P*-value^d^	–	0.523	0.286	–	–	–	–
India (*N* = 372)	Anxiety Mean ± *SD*	1.58 ± 1.791	1.65 ± 1.858	56	69	247	0.284^a^
	*N* (%)	92 (24.73%)	97 (26.1%)	24	29	319	0.583^b^
KSA (*N* = 305)	Anxiety Mean ± *SD*	1.90 ± 1.863	1.67 ± 1.801	83	45	117	**0.002^a^**
	*N* (%)	82 (26.9%)	68 (22.3%)	26	12	267	**0.035^b^**
*P*-value^c^	–	**0.012**	0.662	–	–	–	–
*P*-value^d^	–	0.537	0.281	–	–	–	–
India (*N* = 372)	PTSD Mean ± *SD*	11.56 ± 6.013	10.98 ± 6.635	172	98	102	**<0.001^a^**
KSA (N = 305)		8.73 ± 5.723	8.21 ± 6.117	133	94	78	**0.017^b^**
*P*-value^c^	–	**<0.001**	**<0.001**	–	–	–	–
India (*N* = 372)	Perceptive need for mental health support *N* (%)	161 (43.3%)	161 (43.3%)	19	19	334	1.000^a^
KSA (*N* = 305)		142 (46.6%)	134 (43.9%)	7	15	283	0.134^b^
*P*-value^d^	–	0.437	0.876	–	–	–	–

*^a^Wilcoxon signed rank test; ^b^McNemar test; ^c^Mann Whitney U-test; ^d^Chi-square test; Significant P-values are shown in bold (P < 0.05). KSA, Kingdom of Saudi Arabia.*

### Association Between Sociodemographic Variables and Mental Health Parameters Before and After the Advent of COVID-19 Vaccines

Unadjusted binary logistic regression analysis of mental health parameters with sociodemographic variables as independent variables showed that in the Indian population, educational status (*P* = 0.025) and marital status (*P* = 0.035) was significantly associated with anxiety levels before AC19V, and marital status was significantly associated with perceived need for MHS after AC19V (*P* = 0.048) (Supplementary Tables 2, 4).

In the Saudi population, age was a protective factor for depression, anxiety, and perceived need for MHS before and after AC19V. Gender was significantly associated with anxiety and perceived need for MHS before and after AC19V. Marital status was significantly associated with depression before and after AC19V, and anxiety before AC19V. Place of residence was significantly associated with anxiety and the perceived need for MHS before and after AC19V. Employment status was significantly associated with depression before and after AC19V. Monthly income was significantly associated with anxiety before and after AC19V (Supplementary Tables 6–10).

Cross sectionally, in the Indian population, females had higher scores of depression than males before AC19V, and those without any monthly income had higher scores of depression compared to others both before and after AC19V and higher anxiety scores before AC19V. In the Saudi population, both before and after AC19V, unmarried participants, those residing in rural areas, and students of healthcare professions had higher scores of depression than married individuals, those from urban areas, and those who were unemployed, non-healthcare workers, and healthcare workers, respectively. Anxiety scores were higher in females compared to males, unmarried individuals compared to married ones, participants residing in rural areas compared to those residing in urban areas before and after AC19V and in those without any income compared to others before AC19V (Tables 2–4).

**TABLE 2:** Comparison of depression symptoms stratified by sociodemographic variables before and after the advent of COVID-19 vaccines.

Groups	India				KSA				India vs. KSA	
	*N*	Before (Mean ± SD)	After (Mean ± SD)	*P*-value^a^	*N*	Before (Mean ± *SD*)	After (Mean ± *SD*)	*P*-value^a^	Before *P*-value^c^	After *P*-value^c^
**Gender** Male	135	1.81 ± 1.686	1.91 ± 1.926	0.459	105	2.04 ± 1.792	1.80 ± 1.789	0.247	0.34	0.757
Female	237	2.14 ± 1.643	2.11 ± 1.808	0.580	200	2.41 ± 1.972	2.08 ± 2.059	**0.001**	0.355	0.416
*P*-value^c^		**0.04**	0.175			0.162	0.359			
**Educational status** Postgraduates and higher	33	1.45 ± 1.641	1.39 ± 1.499	0.621	18	1.28 ± 1.074	0.94 ± 0.938	0.196	0.967	0.416
Undergraduates	336	2.07 ± 1.648	2.10 ± 1.871	0.789	218	2.32 ± 1.909	2.02 ± 1.952	**0.009**	0.243	0.472
School level education	3	3.00 ± 3.000	2.67 ± 2.309	0.655	60	2.32 ± 1.961	2.03 ± 2.075	0.141	0.658	0.620
Nil	0	–	–	–	9	3.11 ± 2.619	2.89 ± 2.713	0.157	–	–
*P*-value^e^		0.069	0.110			0.149^e^	0.158^e^			
**Marital status** Single/widowed/divorced	333	2.08 ± 1.671	2.11 ± 1.875	0.779	218	2.61 ± 1.944	2.26 ± 2.018	**0.002**	**0.003**	0.516
Married	39	1.56 ± 1.553	1.46 ± 1.536	0.566	87	1.45 ± 1.576	1.30 ± 1.671	0.292	0.600	0.430
*P*-value^c^		0.070	0.051			**<0.001**	**<0.001**			
**Place of residence** Rural	119	1.85 ± 1.650	2.06 ± 1.945	0.115	53	2.83 ± 2.064	2.62 ± 2.281	0.255	**0.004**	0.169
Urban	253	2.10 ± 1.668	2.03 ± 1.809	0.276	252	2.16 ± 1.869	1.85 ± 1.878	**0.002**	0.971	0.159
*P*-value^c^		0.150	0.934			**0.033**	**0.027**			
**Monthly income** Above 50,000 INR Above 10,000 SAR	25	1.32 ± 1.725	1.04 ± 1.485	0.356	29	1.83 ± 1.794	1.55 ± 1.804	0.279	NA	NA
Below 50,000 INR Below 10,000 SAR	44	1.48 ± 1.548	1.64 ± 1.780	0.612	165	2.16 ± 1.909	1.92 ± 1.969	0.065	NA	NA
Nil	303	2.16 ± 1.650	2.18 ± 1.860	0.921	111	2.58 ± 1.933	2.20 ± 2.008	**0.009**	NA	NA
*P*-value^e^		**0.002**	**0.002**			0.077^e^	0.192^e^			
**Employment status** Healthcare workers	70	1.76 ± 1.756	1.99 ± 2.123	0.161	35	2.11 ± 1.891	1.77 ± 1.734	0.150	0.386	0.967
Students in healthcare profession	220	2.12 ± 1.633	2.07 ± 1.755	0.501	32	3.09 ± 2.006	2.91 ± 2.053	0.273	**0.007**	**0.026**
Non-healthcare workers/unemployed	82	1.98 ± 1.663	1.99 ± 1.876	0.986	238	2.19 ± 1.891	1.89 ± 1.969	**0.005**	0.525	0.531
*P*-value^e^		0.191	0.598			**0.041**	**0.023**			

*^a^Wilcoxon signed rank test; ^c^Mann Whitney U-test; ^e^Kruskal Wallis test; Significant P-values are shown in bold (P < 0.05). KSA; Kingdom of Saudi Arabia, INR; Indian Rupee, SAR; Saudi Riyal.*

**TABLE 3:** Comparison of anxiety symptoms stratified by sociodemographic variables before and after the advent of COVID-19 vaccines.

Groups	India				KSA				India vs. KSA	
	*N*	Before (Mean ± *SD*)	After (Mean ± *SD*)	*P*-value^a^	*N*	Before (Mean ± SD)	After (Mean ± *SD*)	*P*-value ^a^	Before *P*-value^c^	After *P*-value ^c^
**Gender** Male	135	1.49 ± 1.958	1.56 ± 1.965	0.652	105	1.32 ± 1.418	1.24 ± 1.484	0.525	0.579	0.673
Female	237	1.63 ± 1.691	1.71 ± 1.796	0.303	200	2.20 ± 1.997	1.89 ± 1.912	**0.001**	**0.005**	0.373
*P*-value^c^		0.091	0.188			**0.001**	**0.005**			
**Educational status** Postgraduates and higher	33	0.91 ± 1.284	0.97 ± 1.380	0.747	18	0.78 ± 1.060	0.83 ± 0.985	0.792	0.796	0.991
Undergraduates	336	1.63 ± 1.807	1.71 ± 1.890	0.228	218	1.94 ± 1.856	1.64 ± 1.768	**0.001**	**0.022**	0.943
School level education	3	3.33 ± 3.055	2.33 ± 1.528	0.276	60	2.02 ± 1.882	1.90 ± 1.920	0.421	0.399	0.516
Nil	0	NA	NA	NA	9	2.11 ± 2.619	2.44 ± 2.555	0.408	NA	NA
*P*-value^e^		0.061	0.067			0.053	0.195			
**Marital status** Single/widowed/divorced	333	1.64 ± 1.813	1.71 ± 1.895	0.300	218	2.14 ± 1.903	1.86 ± 1.860	**0.001**	**0.001**	0.190
Married	39	1.10 ± 1.535	1.15 ± 1.424	0.772	87	1.29 ± 1.613	1.18 ± 1.552	0.481	0.593	0.948
*P*-value^c^		0.090	0.124			**<0.001**	**0.002**			
**Place of residence** Rural	119	1.51 ± 1.822	1.64 ± 1.903	0.401	53	2.66 ± 2.227	2.36 ± 2.193	0.138	**0.001**	**0.036**
Urban	253	1.61 ± 1.780	1.66 ± 1.840	0.514	252	1.73 ± 1.739	1.52 ± 1.676	**0.006**	0.273	0.648
*P*-value^ c^		0.470	0.769			**0.007**	**0.012**			
**Monthly income** Above 50,000 INR Above 10,000 SAR	25	1.20 ± 1.732	1.04 ± 1.594	0.388	29	1.59 ± 1.842	1.48 ± 1.902	0.709	NA	NA
Below 50,000 INR Below 10,000 SAR	44	0.98 ± 1.422	1.25 ± 1.433	0.179	165	1.66 ± 1.765	1.50 ± 1.724	**0.035**	NA	NA
Nil	303	1.70 ± 1.827	1.76 ± 1.918	0.442	111	2.32 ± 1.945	1.96 ± 1.863	**0.019**	NA	NA
*P*-value^e^		**0.021**	0.070			**0.009**	0.064			
**Employment status** Health professionals	70	1.50 ± 1.886	1.59 ± 1.892	0.724	35	1.71 ± 1.808	1.66 ± 1.679	0.642	0.362	0.505
Students in health profession	220	1.67 ± 1.742	1.66 ± 1.835	0.854	32	2.50 ± 1.984	1.97 ± 1.823	**0.025**	**0.020**	0.219
Non-health professionals/unemployed	82	1.40 ± 1.845	1.70 ± 1.910	0.055	238	1.84 ± 1.846	1.63 ± 1.818	**0.013**	**0.018**	0.911
*P*-value^e^		0.219	0.871			0.127	0.407			

*^a^Wilcoxon signed rank test; ^c^Mann Whitney U-test; ^e^Kruskal Wallis test; Significant P-values are shown in bold (P < 0.05). KSA; Kingdom of Saudi Arabia, INR; Indian Rupee, SAR; Saudi Riyal.*

**TABLE 4:** Comparison of PTSD symptoms stratified by sociodemographic variables before and after the advent of COVID-19 vaccines.

Groups	India				KSA				India vs. KSA	
	*N*	Before (Mean ± SD)	After (Mean ± SD)	*P*-value^a^	*N*	Before (Mean ± SD)	After (Mean ± SD)	*P*-value^a^	Before *P*-value^c^	After *P*-value^c^
**Gender** Male	135	11.68 ± 5.950	10.82 ± 6.486	**0.006**	105	9.06 ± 5.333	8.51 ± 5.997	0.086	**0.001**	**0.008**
Female	237	11.49 ± 6.060	11.07 ± 6.730	**0.018**	200	8.55 ± 5.923	8.05 ± 6.188	0.092	** < 0.001**	**<0.001**
*P*-value ^c^		0.811	0.487			0.336	0.473			
**Educational status** Postgraduates and higher	33	11.67 ± 6.096	11.09 ± 6.079	0.380	18	9.94 ± 4.905	9.00 ± 5.626	0.275	0.343	0.286
Undergraduates	336	11.57 ± 6.032	10.99 ± 6.714	**0.001**	218	8.78 ± 5.911	8.35 ± 6.106	0.086	**<0.001**	**<0.001**
School level education	3	10.00 ± 3.606	8.67 ± 3.786	0.593	60	8.22 ± 5.573	7.37 ± 6.273	0.112	0.539	0.571
Nil	0	NA	NA	NA	9	8.33 ± 3.354	8.89	0.888	NA	NA
*P*-value^e^		0.854	0.793			0.705	0.567			
**Marital status** Single/widowed/divorced	333	11.46 ± 5.994	10.89 ± 6.669	**0.001**	218	8.56 ± 5.630	8.08 ± 6.101	**0.045**	**<0.001**	**<0.001**
Married	39	12.44 ± 6.181	11.77 ± 6.360	0.258	87	9.14 ± 5.963	8.54 ± 6.179	0.159	**0.007**	**0.009**
*P*-value ^c^		0.364	0.409			0.374	0.466			
**Place of residence** Rural	119	11.23 ± 6.296	10.69 ± 6.823	**0.024**	53	9.40 ± 6.090	8.92 ± 6.773	0.501	0.079	0.090
Urban	253	11.72 ± 5.881	11.12 ± 6.553	**0.005**	252	8.59 ± 5.645	8.06 ± 5.973	**0.020**	** < 0.001**	**<0.001**
*P*-value ^c^		0.419	0.680			0.378	0.529			
**Monthly income** Above 50,000 INR Above 10,000 SAR	25	10.28 ± 6.188	8.84 ± 6.681	**0.047**	29	9.97 ± 5.095	9.52 ± 5.026	0.619	NA	NA
Below 50,000 INR Below 10,000 SAR	44	12.30 ± 5.572	12.07 ± 5.683	0.673	165	8.96 ± 6.102	8.10 ± 6.468	**0.006**	NA	NA
Nil	303	11.56 ± 6.061	11.00 ± 6.736	**0.001**	111	8.06 ± 5.240	8.04 ± 5.840	0.715	NA	NA
*P*-value ^e^		0.249	0.097			0.185	0.255			
**Employment status** Health professionals	70	12.30 ± 6.570	12.54 ± 6.909	0.784	35	7.80 ± 5.218	7.43 ± 5.658	0.600	**0.001**	**<0.001**
Students in health profession	220	11.40 ± 5.919	10.45 ± 6.547	**<0.001**	32	8.25 ± 6.720	9.06 ± 7.255	0.614	**0.002**	0.164
Non-health professionals/unemployed	82	11.37 ± 5.790	11.07 ± 6.494	0.449	238	8.93 ± 5.657	8.21 ± 6.027	**0.009**	**0.001**	**0.001**
*P*-value ^e^		0.717	0.134			0.324	0.760			

*^a^Wilcoxon signed rank test; ^c^Mann Whitney U-test; ^e^Kruskal Wallis test; Significant P-values are shown in bold (P < 0.05). KSA; Kingdom of Saudi Arabia, INR; Indian Rupee, SAR; Saudi Riyal.*

Comparison of mental health parameters before and after AC19V showed that there was a significant reduction in the scores of depression after AC19V in females (*P* = 0.001), and those with undergraduate level educational status (*P* = 0.009), unmarried individuals (*P* = 0.002), those residing in urban areas (*P* = 0.002), individuals without income (0.009), and unemployed and those employed in non-healthcare professions (*P* = 0.005) in the Saudi population (Table 2). The scores of anxiety showed a significant reduction in Saudi females (*P* = 0.001), those with undergraduate level educational status (*P* = 0.001), unmarried individuals (*P* = 0.001), those residing in urban areas (*P* = 0.006), those without income (*P* = 0.019), and those with monthly income less than 10,000 SAR (*P* = 0.035), students in healthcare professions (*P* = 0.025) and those who are unemployed and healthcare workers (*P* = 0.013). There were no significant changes in depression and anxiety scores in any of the subgroups of the Indian population in relation to AC19V (Tables 2, 3). The current study found a reduction in the scores for PTSD after AC19V in the Indian population in both males (*P* = 0.006) and females (*P* = 0.018), those with undergraduate level educational status (*P* = 0.001), unmarried individuals (*P* = 0.001), participants residing in both rural (*P* = 0.024) and urban areas (*P* = 0.005), participants with monthly income above 50,000 INR (*P* = 0.047) and those without any income (*P* = 0.001) and in students in healthcare profession (*P* < 0.001) (Table 4). In the case of the Saudi population, the PTSD scores significantly reduced in unmarried individuals (*P* = 0.045), those residing in urban areas (*P* = 0.02), those with monthly income less than 10,000 SAR (*P* = 0.006), and those who were unemployed and non-healthcare workers (*P* = 0.009) after AC19V (Table 4).

### Comparison of Mental Health Parameters Between India and Saudi Arabia

Comparison of scores of depression between the two countries showed that unmarried individuals (*P* = 0.003), those residing in rural areas (*P* = 0.004) before AC19V, and students in healthcare profession (*P* = 0.007, 0.026) before and after AC19V from India had significantly lower levels of depression when compared to their Saudi counterparts (Table 2). In case of anxiety symptoms, females (*P* = 0.005), undergraduates (*P* = 0.022), unmarried individuals (*P* = 0.001), students in the healthcare field (*P* = 0.02), unemployed and non-healthcare workers (*P* = 0.018) before AC19V and rural area residing individuals before (*P* = 0.001) and after (*P* = 0.036) AC19V in India had significantly lower levels of anxiety symptoms when compared to their Saudi counterparts (Table 3).

PTSD scores were significantly higher in Indians before and after AC19V in both males (*P* = 0.001, 0.008) and females (*P* < 0.001), married (*P* = 0.007, 0.009) and unmarried individuals (*P* < 0.001), undergraduates (*P* < 0.001), those residing in urban areas (*P* < 0.001), healthcare workers (*P* = 0.001, *P* < 0.001) and non-healthcare workers and unemployed individuals (*P* = 0.001, 0.001) and before AC19V alone in Indian students in the healthcare field (*P* = 0.002) when compared to the corresponding groups in Saudi population (Table 4).

### Association Between COVID-19 Vaccine Hesitancy and Sociodemographic Variables Between India and Saudi Arabia

Unadjusted binary logistic regression analysis of vaccine hesitancy showed that none of the sociodemographic variables was significantly associated with COVID-19 vaccine hesitancy in India (Supplementary Table 5). In Saudi Arabia, females were found to be more likely to have vaccine hesitancy than males (*P* = 0.039) (Supplementary Table 10).

Vaccine hesitancy was significantly higher in Saudis than in Indians (*P* = 0.001). Within the subgroups, vaccine hesitancy was higher in Saudi females (*P* = 0.002), undergraduates (*P* = 0.004), unmarried individuals (*P* = 0.002), non-healthcare workers and unemployed individuals (*P* = 0.02) and those residing in both urban (*P* = 0.03) and rural areas (*P* = 0.001) when compared to the corresponding Indians. In Saudi Arabia, Vaccine hesitancy was significantly higher in individuals residing in rural areas than those residing in urban areas (*P* = 0.033) (Table 5).

**TABLE 5:** Comparison of COVID-19 vaccine hesitancy scores stratified by sociodemographic variables between India and Saudi Arabia.

	India		KSA		India vs. KSA
	*N*	Mean ± *SD*	*N*	Mean ± *SD*	*P*-value
Overall	372	27.22 ± 5.266	305	29.50 ± 8.569	**0.001^c^**
		189 (50.8%)		170 (55.7%)	0.216^d^
**Gender** Male	135	26.82 ± 5.707	105	28.75 ± 7.949	0.130^c^
Female	237	27.45 ± 4.997	200	29.89 ± 8.871	**0.002^c^**
*P*-value^c^		0.356		0.222	–
**Educational status** Postgraduates and higher	33	26.36 ± 4.974	18	25.78 ± 6.700	0.508^c^
Undergraduates	336	27.32 ± 5.297	218	29.56 ± 8.479	**0.004^c^**
School level education	3	25.00 ± 5.292	60	30.18 ± 9.571	0.286^c^
Nil	0	–	9	31.11 ± 5.600	NA
*P*-value^e^		0.384		0.188	
**Marital status** Single/widowed/divorced	333	27.31 ± 5.307	218	29.71 ± 8.694	**0.002^c^**
Married	39	26.49 ± 4.909	87	28.99 ± 8.275	0.150^c^
*P*-value^c^		0.429		0.509	–
**Place of residence** Rural	119	27.37 ± 5.256	53	31.36 ± 7.913	**0.001** ^c^
Urban	253	27.15 ± 5.280	252	29.11 ± 8.665	**0.030** ^c^
*P*-value^c^		0.547		**0.033**	–
**Monthly income** Above 50,000 INR Above 10,000 SAR	25	25.92 ± 6.370	29	30.03 ± 9.318	NA
Below 50,000 INR Below 10,000 SAR	44	28.16 ± 5.225	165	28.99 ± 8.724	NA
Nil	303	27.19 ± 5.166	111	30.12 ± 8.155	NA
*P*-value^e^		0.340		0.462	
**Employment status** Health professionals	70	28.03 ± 5.321	35	29.51 ± 8.853	0.293^c^
Students in healthcare field	220	27.03 ± 5.261	32	27.31 ± 5.899	0.638^c^
Non-health professionals/unemployed	82	27.04 ± 5.234	238	29.79 ± 8.812	**0.020^c^**
*P*-value^e^		0.344		0.398	

*^c^Mann Whitney U-test; ^e^Kruskal Wallis test; Significant P-values are shown in bold (P < 0.05). ^d^Chi square test; KSA, Kingdom of Saudi Arabia; INR, Indian Rupee; SAR, Saudi Riyal.*

### Correlation Between Mental Health Parameters and COVID-19 Vaccine Hesitancy

COVID-19 vaccine hesitancy was positively correlated with depression and anxiety symptoms before and after AC19V in Saudi Arabia. There was no significant correlation between vaccine hesitancy and any mental health parameters in India (Tables 6, 7).

**TABLE 6:** Correlation between scores in the Indian sample.

Variables	VHS	Depression before	Anxiety before	PTSD before	Depression after	Anxiety after	PTSD after
VHS	1.000	–	–	–	–	–	–
Depression before	−0.003 (0.949)	1.000	–	–	–	–	–
Anxiety before	0.046 (0.377)	**0.608** **(<0.001)**	1.000	–	–	–	–
PTSD before	−0.059 (0.255)	**0.230** **(<0.001)**	**0.352** **(<0.001)**	1.000	–	–	–
Depression after	−0.032 (0.534)	**0.696** **(<0.001)**	**0.634** **(<0.001)**	**0.349** **(<0.001)**	1.000	–	–
Anxiety after	0.067 (0.199)	**0.582** **(<0.001)**	**0.805** **(<0.001)**	**0.400** **(<0.001)**	**0.704** **(<0.001)**	1.000	–
PTSD after	−0.019 (0.709)	**0.273** **(<0.001)**	**0.386** **(<0.001)**	**0.827** **(<0.001)**	**0.416** **(<0.001)**	**0.467** **(<0.001)**	1.000

*The results are expressed as ρ (Rho) value.*

*Significant P-values are shown in bold (P < 0.05).*

**TABLE 7:** Correlation between scores in the K Saudi Arabian sample.

Variables	VHS	Depression before	Anxiety before	PTSD before	Depression after	Anxiety after	PTSD after
VHS	1.000	–	–	–	–	–	–
Depression before	**0.295** **(<0.001)**	1.000	–	–	–	–	–
Anxiety before	**0.258** **(<0.001)**	**0.655** **(<0.001)**	1.000	–	–	–	–
PTSD before	0.043 (0.459)	**0.115** **(0.044)**	**0.259** **(<0.001)**	1.000	–	–	–
Depression after	**0.194** **(0.001)**	**0.680** **(<0.001)**	**0.593** **(<0.001)**	**0.198** **(<0.001)**	1.000	–	–
Anxiety after	**0.277** **(<0.001)**	**0.536** **(<0.001)**	**0.723** **(<0.001)**	**0.353** **(<0.001)**	**0.711** **(<0.001)**	1.000	–
PTSD after	0.044 (0.441)	**0.139** **(0.015)**	**0.296** **(<0.001)**	**0.743** **(<0.001)**	**0.264** **(<0.001)**	**0.383** **(<0.001)**	1.000

*The results are expressed as ρ (Rho) value. Significant P-values are shown in bold (P < 0.05).*

### Adjusted Binary Logistic Regression Analysis of COVID-19 Vaccine Hesitancy

**FIGURE 4:**
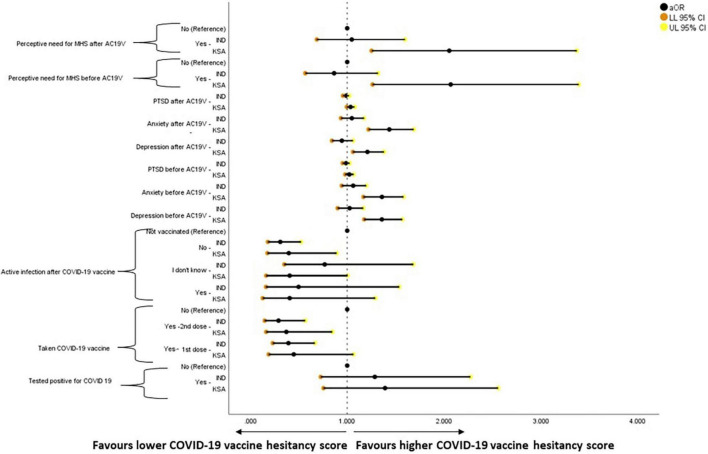
Forest plot showing adjusted binary logistic regression analysis of COVID-19 vaccine hesitancy (regression model 2). aOR, adjusted Odds Ratio; Odds ratio adjusted for Sociodemographic factors. 95% CI—95% Confidence Interval. The results of regression model 1, 2, and 3 in binary logistic regression analysis of COVID-19 Vaccine Hesitancy is tabulated in Supplementary Table 11. Ind–India; KSA–Kingdom of Saudi Arabia.

The binary logistic regression analysis results for COVID-19 vaccine hesitancy are given in Figure 4. Those who had taken COVID-19 vaccine and those who did not develop active infection after COVID-19 vaccinations were less likely to have vaccine hesitancy when compared to those who were not vaccinated in both India and Saudi Arabia. Higher levels of depression, anxiety, and perceived need for MHS before and after AC19V were associated with higher vaccine hesitancy in Saudi Arabia (Figure 3).

### Adjusted Binary Logistic Regression Analysis of Mental Health Parameters

**FIGURE 5:**
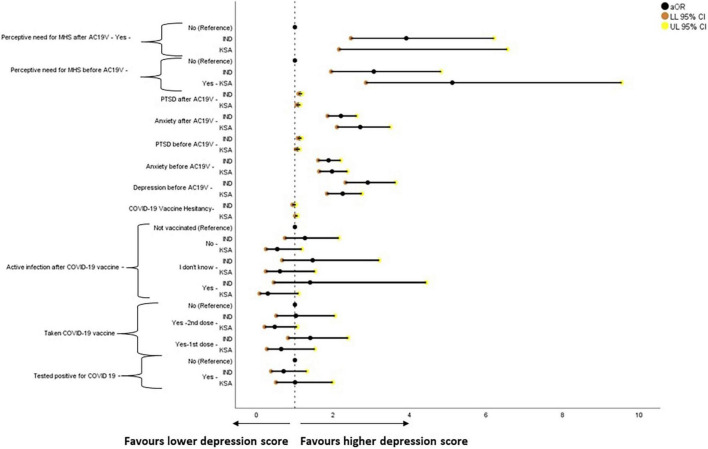
Forest plot showing adjusted binary logistic regression analysis of depression scores (regression model 2). aOR, adjusted Odds Ratio; Odds ratio adjusted for Sociodemographic factors. 95% CI—95% Confidence Interval. The results of regression model 1, 2, and 3 in binary logistic regression analysis of depression scores is tabulated in Supplementary Table 12. Ind–India; KSA–Kingdom of Saudi Arabia.

**FIGURE 6:**
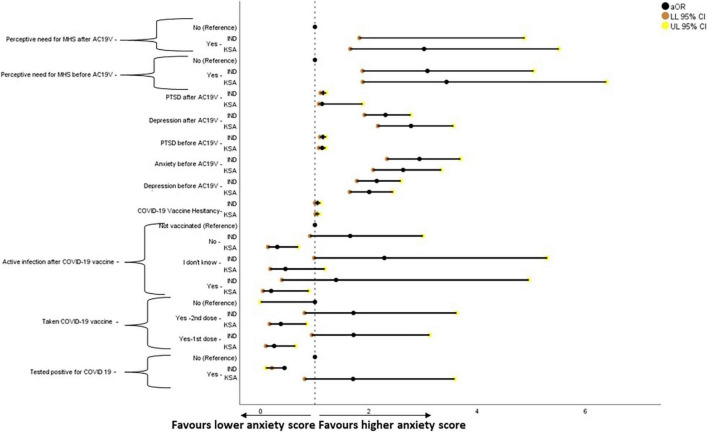
Forest plot showing adjusted binary logistic regression analysis of anxiety scores (REGRESSION model 2). aOR, adjusted Odds Ratio; Odds ratio adjusted for Sociodemographic factors. 95% CI, 95% Confidence Interval. The results of regression model 1, 2, and 3 in binary logistic regression analysis of depression scores is tabulated in Supplementary Table 13. Ind–India; KSA–Kingdom of Saudi Arabia.

**FIGURE 7:**
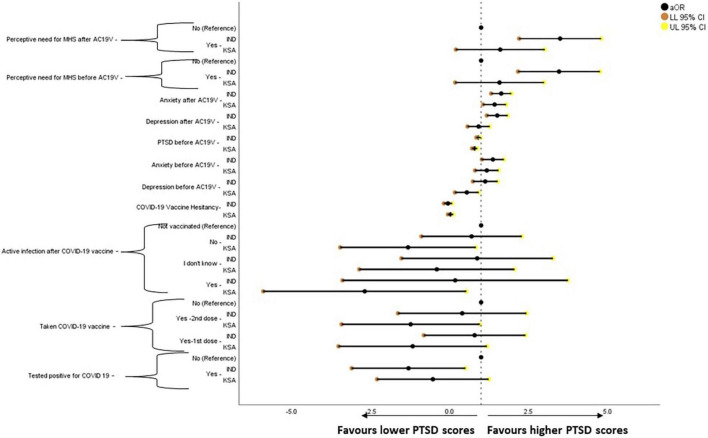
Forest plot showing adjusted generalized linear regression analysis of post-traumatic stress disorder scores (regression model 2). aOR, adjusted Odds Ratio; Odds ratio adjusted for Sociodemographic factors. 95% CI, 95% Confidence Interval. The results of regression model 1, 2, and 3 in binary logistic regression analysis of depression scores is tabulated in Supplementary Table 14. Ind–India; KSA–Kingdom of Saudi Arabia.

**FIGURE 8:**
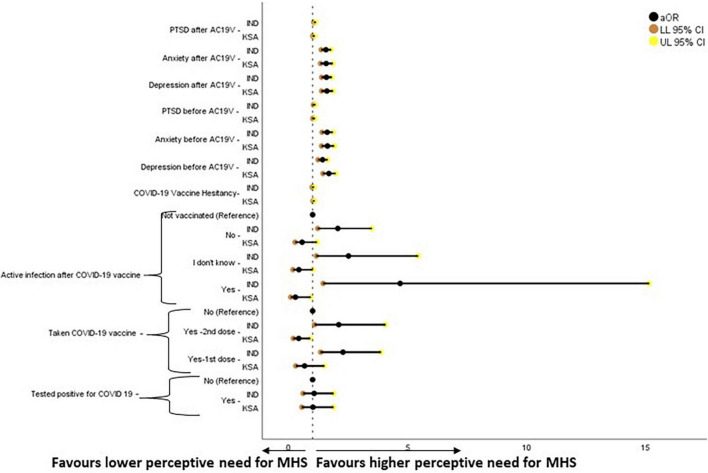
Forest plot showing adjusted binary logistic regression analysis of perception of need for Mental Health Support (regression model 2). aOR, adjusted Odds Ratio; Odds ratio adjusted for Sociodemographic factors. 95% CI, 95% Confidence Interval. The results of regression model 1, 2, and 3 in binary logistic regression analysis of depression scores is tabulated in Supplementary Table 15.

The binary logistic regression analysis results for mental health parameters are given in Figures 5–8. Higher scores of anxiety, PTSD, the perceived need for MHS before and after AC19V, and depression before AC19V were associated with higher scores of depression in both Indian (*P* < 0.001) and Saudi population (*P* < 0.001, *P* = 0.003 for PTSD before). Higher levels of vaccine hesitancy were associated with higher levels of depression (*P* = 0.02) in the Saudi population (Figure 4).

Those who were vaccinated against COVID-19 (*P* = 0.004 -1st dose, *P* = 0.018 -2nd dose) and those who developed active infection after COVID-19 vaccination (*P* = 0.034) and those who did not (*P* = 0.004) were found to be significantly less likely to have anxiety symptoms when compared to those who were not vaccinated against COVID-19 in Saudi Arabia. In India, those who were tested positive for COVID-19 were found to be less likely to have anxiety symptoms (*P* = 0.035). Higher scores of depression, PTSD, and perceived need for MHS before and after AC19V and anxiety before AC19V were significantly associated with higher scores of anxiety in India and Saudi Arabia (*P* < 0.001). Higher scores of vaccine hesitancy were found to be significantly associated with higher levels of anxiety in India (*P* = 0.049) and Saudi Arabia (*P* = 0.009) (Figure 6).

Generalized linear regression analysis of PTSD is given in Figure 6. Higher scores of depression, anxiety, and perceived need for MHS before and after AC19V, and PTSD before AC19V were associated with higher scores of PTSD in India (*P* < 0.001) and Saudi Arabia (*P* < 0.001, *P* = 0.004 for depression before, *P* = 0.027, *P* = 0.025 for perceived need for MHS before and after AC19V) (Figure 7).

Higher scores of depression, anxiety, PTSD before and after AC19V and perceived need for MHS before AC19V were associated with higher perceived need for MHS in India and Saudi Arabia. Higher vaccine hesitancy was associated with the higher perceptive need for MHS in Saudi Arabia. Indians who were vaccinated against COVID-19 and either developed or did not develop an active infection after the vaccination were more likely to have a higher perceived need for MHS. Saudis who had taken the COVID-19 vaccine second dose and those who developed an active infection after the vaccine were less likely to have a higher perceived need for MHS (Figure 8).

## Discussion

The present study investigated the mental health status before and after the advent of COVID-19 vaccines and its association with vaccine hesitancy in the adult population of India and Saudi Arabia. We used a new COVID-19 vaccine hesitancy scale and performed psychometric analysis which showed high validity and reliability in both English and Arabic versions. At the cut off value of 28, the scale demonstrated good sensitivity and moderate specificity (Supplementary Figures 3, 4). The prevalence of depression in India and Saudi Arabia was 36% (95% CI 31–41%) and 38.7% (95% CI 33.22–44.15%) before AC19V and 34.4% (95% CI 29.58–39.24%) and 30.5% (95% CI 25.33–35.66%) after AC19V. The prevalence of anxiety in India and Saudi Arabia was 24.73% (95% CI 20.35–29.12%) and 26.9% (95% CI 21.91–31.86%) before AC19V and 26.1% (95% CI 21.61–30.54%) and 22.3% (95% CI 17.62–26.97%) after AC19V. 43.3% (95% CI 38.24–48.31%) of the Indians expressed the need for mental health support before and after AC19V while 46.6% (95% CI 40.96–52.16%) and 43.9% (95% CI 38.36–49.5%) of Saudis expressed the need for MHS before and after AC19V.

### Mental Health Status

The study found that PTSD symptoms showed a significant reduction in both India and Saudi Arabia after AC19V. However, the prevalence and levels of depression and anxiety symptoms decreased significantly in the Saudi population but not in the Indian population. The anxiety levels were higher in Saudi Arabia than in India before AC19V, but they significantly reduced after AC19V, and levels got almost as same as that of India (Table 2). The possible cause for this could be that Saudi Arabia was more severely affected by the earlier Middle East Respiratory Syndrome (MERS) pandemic in 2012 with 80% of global cases while there was no MERS spread in India (63). Given that there were no vaccines against MERS even till date, it is quite plausible that the Saudi’s symptoms of anxiety and PTSD reduced following the advent of COVID-19 vaccines (64). On the other hand, PTSD scores were higher in Indians than Saudis both before and after AC19V. Though the PTSD symptoms significantly reduced in India after AC19V, they were still higher than that of Saudi Arabia (Table 1). The PTSD symptoms were higher in India than in Saudi Arabia irrespective of gender, marital status, employment status, and in undergraduates and urban dwellers before and after AC19V and in Indian students in the healthcare field before AC19V when compared to their Saudi counterparts (Table 4). We posit that an earlier experience with a pandemic by Saudis would have been responsible for the reduced PTSD symptoms compared to Indians for whom the unprecedented SARS-CoV-2 outbreak to the extent of a pandemic would have been perceived to be comparatively more traumatic. Another reason could be that the study was conducted when both the nations were experiencing the second wave of COVID-19 outbreak, but the second wave’s severity was higher in India than in Saudi Arabia. Thus, despite AC19V the PTSD symptoms were higher in Indians than Saudis due to the second wave’s severity. However, further studies are needed to validate this statement. Similar to our results, a recent multinational study found that country of residence is an important predictor for PTSD during the COVID-19 pandemic (65).

Investigation of the influence of sociodemographic variables on mental health status showed high heterogeneity between India and Saudi Arabia. Age was found to be a significant protective factor against depression, anxiety, and perceived need for MHS both before and after AC19V in Saudi Arabia but not in India. Similarly, a study conducted in the United Kingdom found younger age to predict depression and anxiety, while a study conducted in United States found age to be not associated with mental health status (66, 67). We found that gender was significantly associated with anxiety and perceived need for MHS before and after AC19V in Saudi Arabia, while there was no association for gender with any of the mental health parameters in India. Saudi females were twice as likely to present with anxiety symptoms before [OR 2.740, 95% CI (1.491–5.034)] and after AC19V [OR 2.163, 95% CI (1.152–4.063)] than Saudi males. On the other hand, Saudi females who were 1.691(1.045–2.738) times more likely to perceive the need for MHS before AC19V were found to be 1.842 (1.129–3.003) times more likely to do so after AC19V when compared to Saudi males (Supplementary Tables 7, 9). Marital status was found to be significantly associated with mental health in both countries. In India, unmarried individuals had thrice the risk of having anxiety symptoms before AC19V [OR 3.143, 95% CI (1.086–9.096)] while after AC19V, they were found to be twice as likely to perceive the need for MHS than married ones [OR 2.086, 95% CI (1.005–4.330)] (Supplementary Tables 2, 4). Similar results were observed in Saudis, where unmarried individuals had thrice the risk of having depression symptoms [OR 3.249, 95% CI (1.813–5.820)], twice the risk of showing anxiety symptoms [OR 1.927, 95% CI (1.042–3.562)] before AC19V and twice the risk of showing depression symptoms after AC19V when compared to married individuals [OR 2.204, 95% CI (1.211–4.010)] (Supplementary Tables 6, 7). Educational status was found to be a significant predictor of anxiety symptoms before AC19V in India. Those with a higher level of educational status were found to be less likely to have anxiety symptoms when compared to those with a lower level of educational status [OR 0.032, 95% CI (0.002–0.527)] (Supplementary Table 2). On the contrary, there was no association between educational status and mental health in Saudi Arabia. Place of residence was significantly related to mental health in Saudi Arabia but not in India. Saudis residing in urban areas were less likely to have symptoms of anxiety before [OR 0.440, 95% CI (0.237–0.817)] and after AC19V [OR 0.481, 95% CI (0.252–0.919)] while also being less likely to perceive the need for MHS both before [OR 0.419, 95% CI (0.227–0.775)] and after [OR 0.491, 95% CI (0.269–0.895)] AC19V when compared with those residing in rural areas. Economic status was a predictor of negative mental health in Saudi Arabia. Saudis with monthly income less than 10,000 SAR was found to be less likely to have symptoms of anxiety before [OR 0.444, 95% CI (0.258–0.764)] and after AC19V [OR 0.483, 95% CI (0.272–0.859)] when compared to those without any income (Supplementary Table 7). Employment status significantly predicted negative mental health in Saudi Arabia but not in India. Saudi students in the healthcare field were three times more likely to have symptoms of depression before [OR 2.841, 95% CI (1.325–6.090)] and after AC19V [OR 3.281, 95% CI (1.545–6.970)] when compared to non-healthcare workers and unemployed individuals (Supplementary Table 6). Our results were consistent with similar studies conducted in other countries, which assessed the relationship between sociodemographic variables and mental health during the COVID-19 pandemic (65–75).

Comparison of the influence of sociodemographic variables on mental health before and after AC19V showed an interesting pattern. PTSD symptoms showed significant reduction after AC19V in both Indian males (*P* = 0.006) and females (*P* = 0.018) irrespective of gender. On the other hand, the levels of anxiety in Saudi females, which were higher than that of Indian females before AC19V (*P* = 0.005), showed a significant reduction after AC19V (*P* = 0.001) and became closer to that of the Indian females (Table 3). Thus, the females who were at a higher risk of developing anxiety symptoms were the ones who responded well to the advent of COVID-19 vaccines. Despite the reduction in anxiety symptoms, Saudi females were still at a higher risk of having anxiety symptoms, albeit with a minor reduction in the odds after AC19V (Figure 6). However, they were also found to have a higher perceived need for MHS than Saudi males, which is an essential step in seeking mental health support (Figure 8). In our study, there was a significant reduction in depression (*P* = 0.002), anxiety (*P* = 0.001), and PTSD symptoms (*P* = 0.45) in unmarried Saudis and a reduction in PTSD symptoms in unmarried Indians (*P* = 0.001) after AC19V (Tables 2–4). Thus, unmarried individuals who were more at risk of developing depression, anxiety and PTSD during the pandemic were also the ones who fared well with the advent of COVID-19 vaccine (Supplementary Tables 1–3). Saudi undergraduates showed a significant reduction in depression (*P* = 0.009) and anxiety (*P* = 0.001) symptoms after AC19V (Tables 2, 3), and Indian undergraduates showed a significant reduction in PTSD symptoms after AC19V (Table 4). Those with lower educational status were at higher risk of negative mental health, and it was those with undergraduate level of education who showed improvement in their mental health with AC19V (Supplementary Tables 1–4, 6–9). Depression (*P* = 0.002), anxiety (*P* = 0.006), and PTSD (*P* = 0.020) levels of Saudis living in urban areas decreased with AC19V. Saudis in rural areas had higher depression levels (*P* = 0.004) before AC19V and higher anxiety levels before (*P* = 0.001) and after (*P* = 0.036) AC19V than Indian rural dwellers. PTSD levels of Indians residing in both urban (*P* = 0.005) and rural areas (*P* = 0.024) decreased after AC19V. The influence of place of residence on mental health in relation to AC19V was contradictory to other sociodemographic variables. Urban dwellers who were less vulnerable to the negative impact of the pandemic on mental health showed significant improvement with AC19V. Saudis without any income showed a significant reduction in depression (*P* = 0.009) and anxiety (*P* = 0.019) symptoms after AC19V. PTSD symptoms significantly decreased in Indians with monthly income above 50,000 INR (*P* = 0.047) and those without any income (*P* = 0.001) and in Saudis with income less than 10,000 SAR (*P* = 0.006) after AC19V. Even though the changes in PTSD symptoms showed heterogeneity in relation to economic status, depression and anxiety were reduced in the no-income group with the advent of the COVID-19 vaccine which was the high-risk group. Saudi non-healthcare workers and unemployed individuals showed a significant reduction in depression (*P* = 0.005), anxiety (*P* = 0.013) and PTSD symptoms (*P* = 0.009) after AC19V. Saudi students in the healthcare field showed a significant reduction in anxiety symptoms (*P* = 0.025) after AC19V. Saudi students in the healthcare field had higher levels of depression symptoms when compared to those in India both before (*P* = 0.007) and after AC19V (*P* = 0.026). Anxiety levels were higher in Saudi students in the healthcare field (*P* = 0.020) and non-healthcare workers and unemployed individuals (*P* = 0.018) when compared to the corresponding subset in India before AC19V (Tables 2–4). The response of the study population to AC19V in both countries showed heterogeneity in relation to their employment status, wherein a reduction in negative mental health symptoms was observed irrespective of the risk of negative mental health before AC19V. Thus, except for the place of residence and employment status, those in the subgroups of sociodemographic variables who were at higher risk of negative mental health before AC19V were the ones who showed improvement in their mental health after AC19V.

### COVID-19 Vaccine Hesitancy

The prevalence of COVID-19 vaccine hesitancy in India was 50.8% (95% CI 45.73–55.89%) and in Saudi Arabia was 55.7% (95% CI 50.16–61.31%). Though the percentage of the study participants who were not vaccinated against COVID-19 was less (26% in India and 12.1% in Saudi Arabia), vaccine hesitancy was relatively higher. This shows that even those who got themselves vaccinated against COVID-19 continued to exhibit vaccine hesitancy. Similar results were observed in a study conducted in Israel (76). Lack of data availability regarding the long term effects of the vaccine and the general mistrust regarding its efficacy and safety could be the reasons behind this finding (77). Comparison of vaccine hesitancy between the two countries showed that the levels of vaccine hesitancy were significantly higher in Saudi Arabia than in India though there was no difference in their prevalence (Table 5). In Saudi Arabia, among the sociodemographic variables, place of residence and gender was found to be significantly associated with vaccine hesitancy. Females were 1.65 (95% CI: 1.025–2.656) times more likely to have vaccine hesitancy than males (Supplementary Table 10) and people living in rural areas had higher vaccine hesitancy than those living in urban areas (Table 5). Similar to our results, globally, females have been found to be more vaccine hesitant than males (78). One possible reason could be that females who were pregnant and lactating were excluded from most COVID-19 vaccine clinical trials, and this would not have been reassuring for this subset of women and to those who were trying to get pregnant. Regarding the relation between the place of residence and vaccine hesitancy, the results in other studies vary from no relation (79) to higher vaccine hesitancy in rural area dwellers than urban area dwellers (80, 81). With urban areas being the central hub of activities with higher population size and hence increased disease spread, the rural area dwellers might have felt relatively safer and not compelled to get vaccinated. On the other hand, there was no significant association between any of the studied sociodemographic variables and vaccine hesitancy in India. This finding is in contrast to the study conducted in June 2021 in India, which found age and gender to be significantly related to vaccine hesitancy (82). However, similar to our results, other multinational studies assessing vaccine hesitancy has found the association between sociodemographic variables and vaccine hesitancy to be varying in different countries (83).

Comparison of vaccine hesitancy between India and Saudi Arabia showed that the levels of vaccine hesitancy were higher in Saudi Arabia than in India and in Saudi females, unmarried individuals, those with undergraduate level of educational status, non-healthcare workers and unemployed individuals and those residing in urban and rural areas when compared to their Indian counterparts. A recent multinational study conducted by Qunaibi et al. (84) in 23 Arab countries and 122 non-Arab countries has found that willingness to vaccinate was higher in countries with higher rates of COVID-19 (84). This explains the higher COVID-19 vaccine hesitancy in Saudi Arabia than India, which had lesser severity of COVID-19 spread than India at the time of the survey.

Analysis of risk and protective factors for vaccine hesitancy showed that above and beyond the effect of sociodemographic factors, COVID-19 status of being vaccinated and not developing an active infection after vaccination was significantly associated with vaccine hesitancy. Being vaccinated against COVID-19 was associated with lower levels of vaccine hesitancy in India and Saudi Arabia. In India, being vaccinated with first [aOR 0.393, 95% CI (0.232–0.666)] and second dose [aOR 0.291, 95% CI (0.149–0.565)] was found to be protective while in Saudi Arabia, being vaccinated with second dose [aOR 0.372, 95% CI (0.164–0.845)] alone was protective against vaccine hesitancy. In contrast, being vaccinated with first dose when adjusted for the effect of confounding sociodemographic variables showed no significant relation (Supplementary Table 11 and Figure 4). Those who did not develop an active infection after COVID-19 vaccinations were found to be less likely to be vaccine-hesitant than those who were not vaccinated both in India [aOR 0.309, 95% CI (0.182–0.522)] and Saudi Arabia [aOR 0.397, 95% CI (0.177–0.890)]. With the COVID-19 vaccine’s safety and efficacy being identified as some of the top reasons for vaccine hesitancy the absence of active infection after vaccination would have been reassuring and favored vaccine acceptance (85, 86).

### COVID-19 Vaccine Hesitancy and Mental Health

The study found a bidirectional association between COVID-19 vaccine hesitancy and mental health in Saudi Arabia, over and above the effect of sociodemographic factors and COVID-19 status in relation to infection and vaccination. Higher levels of vaccine hesitancy were found to increase the risk of depression [aOR 1.033, 95% CI (1.001–1.067)], anxiety [aOR 1.037, 95% CI (1.002–1.074)] and perceived need for MHS [aOR 1.043, 95% CI (1.012–1.075)] (Supplementary Tables 12, 13, 15 and Figures 4, 5, 7). On the other hand, depression [aOR 1.350, 95% CI (1.167–1.563)—before AC19V, aOR 1.200, 95% CI (1.050–1.372)—after AC19V], anxiety [aOR 1.344, 95% CI (1.150–1.570)—before Ac19V, aOR 1.409, 95% CI (1.197–1.659)—after AC19V], and perceived need for MHS [aOR 2.053, 95% CI (1.239–3.403)—before AC19V, aOR 1.958, 95% CI (1.184–3.238)—after AC19V] both before and after AC19V were found to be significant risk factors for vaccine hesitancy with higher levels of these variables favoring higher vaccine hesitancy (Supplementary Table 11 and Figure 4). Contrarily in India, we did not find a bidirectional association between mental health and vaccine hesitancy. None of the mental health parameters was found to predict vaccine hesitancy individually or when adjusted for sociodemographic factors and sociodemographic factors along with COVID-19 status (Supplementary Table 11). Notwithstanding, higher levels of vaccine hesitancy was found to increase the risk for anxiety [aOR 1.058, 95% CI (1.007–1.111)] (Supplementary Table 13 and Figure 5). Similarly, a study done in vaccinated individuals showed that vaccine hesitancy increased the risk for depression, anxiety and peritraumatic stress (76). The present study is the first of its kind to highlight the mutual impact of mental health status and vaccine hesitancy in the general population. Most studies assessing the relation between vaccine hesitancy and mental health were conducted in people with existing mental health disorders. A study conducted in the United Kingdom found that diagnosis of anxiety and depression before the pandemic was not associated with vaccine hesitancy (87). Another study conducted on patients with psychiatric disorders found that generalized anxiety disorder, PTSD and major depressive disorder were not related to vaccine hesitancy once adjusted for sociodemographic factors and physical co-morbidities (88). Thus, the present study gives important insights into the mental health status and its association with vaccine hesitancy in the general population which indicates that the issue of vaccine hesitancy should be addressed immediately to mitigate its effect on mental health.

## Strengths and Limitations of the Study

The study investigated the relationship between mental health status and COVID-19 vaccine hesitancy before and after the advent of COVID-19 vaccines in the general population of India and Saudi Arabia. We used a newly constructed COVID19-VHS12 scale and performed psychometric analysis and validated the scale in English and Arabic versions which enabled us to use it as a binary response scale. The present study is the first to explore the relation between COVID-19 vaccine hesitancy and mental health during the COVID-19 pandemic in the general population and compare between two countries. The exhaustive analysis of the confounders and predictor variables with respect to the advent of the COVID-19 vaccine had enabled us to assert the pattern and delineate the temporal order of the influence of each predictor variable. The comparative study between the two countries will help better understand the varying relation between vaccine hesitancy and mental health across different sociodemographic groups. This will help the healthcare authorities and policymakers devise strategies and policies to surmount the impact of vaccine hesitancy and the negative impact of the pandemic on mental health.

The study is not without shortcomings. The relatively smaller sample size is the main limitation of our study, though the detailed analysis of the collected data outweighs any frailty that may have arisen with the smaller sample. However, the findings of our study should be generalized with caution as the representativeness of the samples is limited. Due to the online nature of the survey, the study participants were primarily from those who had special keenness to know about COVID-19 pandemic and vaccination. Hence, more samples were drawn from healthcare sector and of younger age groups. Another limitation of this study is the use of mental health screening tools, which included ultrashort screening tools, viz., PHQ-2, GAD-2, and IES-6 which cannot substitute a complete clinical examination to arrive at a diagnosis. The cross-sectional nature of the study limits the determination of causality. Given the survey nature of the study, social desirability bias and recall bias to answer the questionnaires could be other limitations of our study.

## Conclusion

COVID-19 vaccine hesitancy is a critical barrier in accomplishing herd immunity against COVID-19. From the results of our study, it is clear that vaccine hesitancy has a negative impact on mental health and vice versa over and above the impact of sociodemographic factors and COVID-19 vaccination and infection status. We demonstrated that the mutual impact of COVID-19 vaccine hesitancy and mental health varied between India and Saudi Arabia which differed in pandemic severity and vaccine mandates. Our study also shows that, vaccine hesitancy is a predictor for depression, anxiety, post-traumatic stress disorder and perceptive need for MHS in Saudi Arabia while, vaccine hesitancy is a predictor for anxiety alone in India. Similarly, all the above-mentioned mental health parameters were predictors of Vaccine hesitancy in Saudi Arabia but not in India. This is a significant finding of this preliminary comparative study which emphasizes variation of mutual impact between vaccine hesitancy and mental health across different borders globally. Future multinational studies are needed to probe further into this phenomenon to devise strategies to address them and better equip vulnerable nations to combat this serious global health threat of vaccine hesitancy.

## Data Availability Statement

The raw data supporting the conclusions of this article will be made available by the authors, without undue reservation, upon reasonable request.

## Ethics Statement

The studies involving human participants were reviewed and approved by the Majmaah University Research Ethics Committee and Institutional Ethical Committee of Madha Medical College and Research Institute. The patients/participants provided their written informed consent to participate in this study.

## Author Contributions

SJ and SI: study conception. SJ, SI, and SK: study design. SK, AP, SM, AAla, AAlj, ASA, and YH: data collection. SJ, SI, AP, SM, AAla, and AAlj: data analysis and manuscript drafting. SJ, SI, AAla, AAlj, ASA, and YH: data interpretation. AP, SM, AAla, and AAlj: critical revision of the manuscript. All authors approved the final version, contributed to the article, and approved the submitted version.

## Conflict of Interest

The authors declare that the research was conducted in the absence of any commercial or financial relationships that could be construed as a potential conflict of interest.

## Publisher’s Note

All claims expressed in this article are solely those of the authors and do not necessarily represent those of their affiliated organizations, or those of the publisher, the editors and the reviewers. Any product that may be evaluated in this article, or claim that may be made by its manufacturer, is not guaranteed or endorsed by the publisher.

## Footnotes

1
http://sampsize.sourceforge.net/iface/
