# Supplementary material for: Contrasting Association Between COVID-19 Vaccine Hesitancy and Mental Health Status in India and Saudi Arabia—A Preliminary Evidence Collected During the Second Wave of COVID-19 Pandemic
Source: Front Med (Lausanne). 2022 May 4;9:900026. doi: 10.3389/fmed.2022.900026 (PMC9116149; doi:10.3389/fmed.2022.900026)
Supplement: Supplementary file 1 [file Data_Sheet_1.docx]

Supplementary Material

# Supplementary Tables

| Variables | Sub-Categories | Before AC19V | | | | After AC19V | | | |
| --- | --- | --- | --- | --- | --- | --- | --- | --- | --- |
|  |  | Crude OR | 95% CI | | P Value | Crude OR | 95% CI | | P Value |
|  |  |  | Lower | Upper |  |  | Lower | Upper |  |
| Age | - | 0.986 | 0.955 | 1.019 | 0.401 | 0.988 | 0.956 | 1.021 | 0.471 |
| Gender | Male* |  |  |  | - |  |  |  | - |
|  | Female | 1.334 | 0.853 | 2.085 | 0.207 | 1.196 | 0.764 | 1.874 | 0.434 |
| Educational status | Postgraduates and higher | 0.135 | 0.011 | 1.709 | 0.122 | 0.135 | 0.011 | 1.709 | 0.122 |
|  | Undergraduates | 0.296 | 0.027 | 3.300 | 0.323 | 0.274 | 0.025 | 3.055 | 0.293 |
|  | School level education* |  |  |  | 0.118 |  |  |  | 0.150 |
|  | Nil | - | - | - | - | - | - | - | - |
| Marital status | Married* |  |  |  | - |  |  |  | **-** |
|  | Unmarried | 1.301 | 0.636 | 2.661 | 0.471 | 1.592 | 0.750 | 3.380 | 0.226 |
| Current residence | Rural* |  |  |  | - |  |  |  | - |
|  | Urban | 1.168 | 0.739 | 1.846 | 0.507 | 0.894 | 0.567 | 1.411 | 0.631 |
| Monthly Income | More than 50,000 INR | 0.502 | 0.195 | 1.294 | 0.154 | 0.439 | 0.160 | 1.201 | 0.109 |
|  | Less than 50,000 INR | 0.530 | 0.258 | 1.089 | 0.084 | 0.736 | 0.370 | 1.465 | 0.383 |
|  | Nil* |  |  |  | 0.097 |  |  |  | 0.209 |
| Employment status | Healthcare workers | 1.204 | 0.617 | 2.349 | 0.587 | 0.904 | 0.464 | 1.763 | 0.768 |
|  | Students in healthcare profession | 1.187 | 0.695 | 2.029 | 0.531 | 0.879 | 0.517 | 1.492 | 0.632 |
|  | Non-healthcare workers/ unemployed* |  |  |  | 0.803 |  |  |  | 0.891 |

**Supplementary table S1:** Binary logistic regression for depression before and after AC19V with Sociodemographic variables as independent variables for the Indian population. OR Odds ratio; 95% CI 95% confidence interval; AC19V advent of COVID-19 vaccine; * Reference category

| Variables | Sub-Categories | Before AC19V | | | | After Ac19V | | | |
| --- | --- | --- | --- | --- | --- | --- | --- | --- | --- |
|  |  | Crude OR | 95% CI | | P Value | Crude OR | 95% CI | | P Value |
|  |  |  | Lower | Upper |  |  | Lower | Upper |  |
| Age | - | 0.967 | 0.928 | 1.009 | 0.122 | 0.977 | 0.940 | 1.015 | 0.235 |
| Gender | Male* |  |  |  | - |  |  |  | - |
|  | Female | 1.091 | 0.666 | 1.786 | 0.729 | 1.295 | 0.792 | 2.117 | 0.303 |
| Educational status | Postgraduates and higher | 0.032 | 0.002 | 0.527 | **0.016** | 0.276 | 0.020 | 3.782 | 0.335 |
|  | Undergraduates | 0.177 | 0.016 | 1.981 | 0.160 | 0.754 | 0.068 | 8.416 | 0.819 |
|  | School level education* |  |  |  | **0.025** |  |  |  | 0.178 |
|  | Nil |  |  |  |  |  |  |  |  |
| Marital status | Married* |  |  |  | - |  |  |  | **-** |
|  | Unmarried | 3.143 | 1.086 | 9.096 | **0.035** | 1.693 | 0.722 | 3.973 | 0.226 |
| Current residence | Rural* |  |  |  | - |  |  |  |  |
|  | Urban | 0.963 | 0.582 | 1.593 | 0.883 | 0.829 | 0.508 | 1.352 | 0.452 |
| Monthly Income | More than 50,000 INR | 0.368 | 0.107 | 1.261 | 0.111 | 0.513 | 0.171 | 1.540 | 0.234 |
|  | Less than 50,000 INR | 0.510 | 0.219 | 1.189 | 0.119 | 0.898 | 0.434 | 1.860 | 0.773 |
|  | Nil* |  |  |  | 0.096 |  |  |  | 0.484 |
| Employment status | Healthcare workers | 1.043 | 0.477 | 2.279 | 0.916 | 1.026 | 0.506 | 2.083 | 0.943 |
|  | Students in healthcare profession | 1.434 | 0.778 | 2.641 | 0.248 | 0.834 | 0.471 | 1.478 | 0.535 |
|  | Non-healthcare workers/ unemployed* |  |  |  | 0.393 |  |  |  | 0.720 |

Supplementary table S2: Binary logistic regression for anxiety before and after AC19V with Sociodemographic variables as independent variables for the Indian population. OR Odds ratio; 95% CI 95% confidence interval; AC19V advent of COVID-19 vaccine; * Reference category

| Variables | Sub-Categories | Before AC19V | | | | After Ac19V | | | |
| --- | --- | --- | --- | --- | --- | --- | --- | --- | --- |
|  |  | B value | 95% CI | | P Value | B value | 95% CI | | P Value |
|  |  |  | Lower | Upper |  |  | Lower | Upper |  |
| Age | - | 0.019 | -0.070 | 0.108 | 0.674 | 0.000441 | -0.099 | 0.098 | 0.993 |
| Gender | Male* | - | - | - | - | - | - | - | - |
|  | Female | -0.188 | -1.457 | 1.081 | 0.772 | 0.250 | -1.151 | 1.650 | 0.727 |
| Educational status | Postgraduates and higher | 1.667 | -5.428 | 8.762 | 0.645 | 2.424 | -5.403 | 10.251 | 0.544 |
|  | Undergraduates | 1.565 | -5.258 | 8.389 | 0.653 | 2.324 | -5.203 | 9.852 | 0.545 |
|  | School level education* | - | - | - | - | - | - | - | - |
|  | Nil |  |  |  |  |  |  |  |  |
| Marital status | Married* | - | - | - | - | - | - | - | - |
|  | Unmarried | -0.976 | -2.966 | 1.013 | 0.336 | -0.880 | -3.076 | 1.316 | 0.432 |
| Current residence | Rural* |  |  |  |  |  |  |  |  |
|  | Urban | 0.492 | -0.815 | 1.800 | 0.460 | 0.430 | -1.013 | 1.872 | 0.560 |
| Monthly Income | More than 50,000 INR* | - | - | - | - | - | - | - | - |
|  | Less than 50,000 INR | 2.015 | -0.925 | 4.956 | 0.179 | 3.228 | -0.008 | 6.464 | 0.051 |
|  | Nil | 1.281 | -1.162 | 3.724 | 0.304 | 2.160 | -0.528 | 4.848 | 0.115 |
| Employment status | Healthcare workers | 0.934 | -0.978 | 2.846 | 0.338 | 1.470 | -0.628 | 3.568 | 0.170 |
|  | Students in healthcare profession | 0.034 | -1.486 | 1.554 | 0.965 | -0.623 | -2.291 | 1.045 | 0.464 |
|  | Non-healthcare workers/ unemployed* | - | - | - | - | - | - | - | - |

Supplementary table S3: Generalized linear regression analysis for PTSD before and after AC19V with Sociodemographic variables as independent variables for the Indian population. OR Odds ratio; 95% CI 95% confidence interval; AC19V advent of COVID-19 vaccine; * Reference category

| Variables | Sub-Categories | Before AC19V | | | | After Ac19V | | | |
| --- | --- | --- | --- | --- | --- | --- | --- | --- | --- |
|  |  | Crude OR | 95% CI | | P Value | Crude OR | 95% CI | | P Value |
|  |  |  | Lower | Upper |  |  | Lower | Upper |  |
| Age | - | 0.994 | 0.964 | 1.024 | 0.687 | 0.980 | 0.950 | 1.012 | 0.218 |
| Gender | Male* |  |  |  | - |  |  |  | - |
|  | Female | 1.177 | 0.767 | 1.806 | 0.456 | 1.177 | 0.767 | 1.806 | 0.456 |
| Educational status | Postgraduates and higher | 0.286 | 0.023 | 3.491 | 0.327 | 0.217 | 0.018 | 2.682 | 0.234 |
|  | Undergraduates | 0.389 | 0.035 | 4.330 | 0.442 | 0.398 | 0.036 | 4.436 | 0.454 |
|  | School level education* |  |  |  | 0.525 |  |  |  | 0.226 |
|  | Nil |  |  |  |  |  |  |  |  |
| Marital status | Married* |  |  |  | - |  |  |  | **-** |
|  | Unmarried | 1.411 | 0.709 | 2.811 | 0.327 | 2.086 | 1.005 | 4.330 | **0.048** |
| Current residence | Rural* |  |  |  | - |  |  |  | - |
|  | Urban | 0.928 | 0.598 | 1.439 | 0.737 | 1.079 | 0.694 | 1.676 | 0.736 |
| Monthly Income | More than 50,000 INR | 0.841 | 0.366 | 1.931 | 0.683 | 0.691 | 0.296 | 1.612 | 0.392 |
|  | Less than 50,000 INR | 0.794 | 0.415 | 1.518 | 0.485 | 0.702 | 0.365 | 1.350 | 0.289 |
|  | Nil* |  |  |  | 0.739 |  |  |  | 0.426 |
| Employment status | Healthcare workers | 1.033 | 0.536 | 1.991 | 0.923 | 1.300 | 0.677 | 2.497 | 0.431 |
|  | Students in healthcare profession | 1.448 | 0.862 | 2.434 | 0.162 | 1.471 | 0.873 | 2.479 | 0.147 |
|  | Non-healthcare workers/ unemployed* |  |  |  | 0.253 |  |  |  | 0.348 |

Supplementary table S4: Binary logistic regression for perceptive need for mental health support before and after AC19V with Sociodemographic variables as independent variables for the Indian population. OR Odds ratio; 95% CI 95% confidence interval; AC19V advent of COVID-19 vaccine; * Reference category

| Variables | Sub-Categories | After AC19V | | | |
| --- | --- | --- | --- | --- | --- |
|  |  | Crude OR | 95% CI | | P Value |
|  |  |  | Lower | Upper |  |
| Age | - | 0.976 | 0.946 | 1.006 | 0.115 |
| Gender | Male* | - | - | - | - |
|  | Female | 1.128 | 0.739 | 1.722 | 0.577 |
| Educational status | Postgraduates and higher | 1.143 | 0.094 | 13.965 | 0.917 |
|  | Undergraduates | 2.200 | 0.198 | 24.494 | 0.521 |
|  | School level education* | - | - | - | - |
|  | Nil |  |  |  |  |
| Marital status | Married* | - | - | - | - |
|  | Unmarried | 1.383 | 0.709 | 2.698 | 0.342 |
| Current residence | Rural* | - | - | - | - |
|  | Urban | 1.023 | 0.662 | 1.582 | 0.919 |
| Monthly Income | More than 50,000 INR | 0.537 | 0.230 | 1.253 | 0.150 |
|  | Less than 50,000 INR | 1.256 | 0.664 | 2.377 | 0.483 |
|  | Nil* | - | - | - | - |
| Employment status | Healthcare workers | 1.270 | 0.669 | 2.412 | 0.465 |
|  | Students in healthcare profession | 0.902 | 0.543 | 1.498 | 0.690 |
|  | Non-healthcare workers/ unemployed* | - | - | - | - |

Supplementary table S5: Binary logistic regression for COVID-19 vaccine hesitancy with Sociodemographic variables as independent variables for the Indian population. OR Odds ratio; 95% CI 95% confidence interval; AC19V advent of COVID-19 vaccine; * Reference category

| Variables | Sub-Categories | Before AC19V | | | | After Ac19V | | | |
| --- | --- | --- | --- | --- | --- | --- | --- | --- | --- |
|  |  | Crude OR | 95% CI | | P Value | Crude OR | 95% CI | | P Value |
|  |  |  | Lower | Upper |  |  | Lower | Upper |  |
| Age | - | 0.937 | 0.908 | 0.968 | **<0.001** | 0.963 | 0.934 | 0.993 | **0.015** |
| Gender | Male* |  |  |  |  |  |  |  |  |
|  | Female | 1.105 | 0.679 | 1.798 | 0.688 | 0.935 | 0.561 | 1.559 | 0.797 |
| Educational status | Postgraduates and higher | 0.100 | 0.014 | 0.719 | **0.022** | <0.001 | <0.001 | <0.001 | 0.998 |
|  | Undergraduates | 0.542 | 0.141 | 2.073 | 0.370 | 0.370 | 0.096 | 1.423 | 0.148 |
|  | School level education | 0.497 | 0.121 | 2.045 | 0.333 | 0.371 | 0.089 | 1.538 | 0.172 |
|  | Nil* |  |  |  | 0.117 |  |  |  | 0.549 |
| Marital status | Married* |  |  |  |  |  |  |  |  |
|  | Unmarried | 3.249 | 1.813 | 5.820 | **<0.001** | 2.204 | 1.211 | 4.010 | **0.010** |
| Current residence | Rural* |  |  |  |  |  |  |  |  |
|  | Urban | 0.597 | 0.329 | 1.084 | 0.090 | 0.553 | 0.300 | 1.019 | 0.057 |
| Monthly Income | More than 10,000 SAR | 0.666 | 0.284 | 1.562 | 0.350 | 0.732 | 0.296 | 1.807 | 0.498 |
|  | Less than 10,000 SAR | 0.704 | 0.431 | 1.151 | 0.162 | 0.765 | 0.456 | 1.284 | 0.311 |
|  | Nil* |  |  |  | 0.333 |  |  |  | 0.560 |
| Employment status | Healthcare workers | 0.682 | 0.313 | 1.486 | 0.335 | 0.756 | 0.327 | 1.748 | 0.513 |
|  | Students in healthcare profession | 2.841 | 1.325 | 6.090 | **0.007** | 3.281 | 1.545 | 6.970 | **0.002** |
|  | Non-healthcare workers/ unemployed* |  |  |  | **0.012** |  |  |  | **0.005** |

Supplementary table S6: Binary logistic regression for depression before and after AC19V with Sociodemographic variables as independent variables for the Saudi Arabian population. OR Odds ratio; 95% CI 95% confidence interval; AC19V advent of COVID-19 vaccine; * Reference category

| Variables | Sub-Categories | Before AC19V | | | | After Ac19V | | | |
| --- | --- | --- | --- | --- | --- | --- | --- | --- | --- |
|  |  | Crude OR | 95% CI | | P Value | Crude OR | 95% CI | | P Value |
|  |  |  | Lower | Upper |  |  | Lower | Upper |  |
| Age | - | 0.956 | 0.924 | 0.988 | **0.008** | 0.961 | 0.927 | 0.995 | **0.027** |
| Gender | Male* |  |  |  |  |  |  |  |  |
|  | Female | 2.740 | 1.491 | 5.034 | **0.001** | 2.163 | 1.152 | 4.063 | **0.016** |
| Educational status | Postgraduates and higher | 0.156 | 0.022 | 1.123 | 0.065 | 0.074 | 0.007 | 0.817 | **0.034** |
|  | Undergraduates | 0.453 | 0.118 | 1.746 | 0.250 | 0.362 | 0.094 | 1.402 | 0.141 |
|  | School level education | 0.536 | 0.129 | 2.230 | 0.391 | 0.380 | 0.090 | 1.613 | 0.190 |
|  | Nil* |  |  |  | 0.296 |  |  |  | 0.196 |
| Marital status | Married* |  |  |  |  |  |  |  |  |
|  | Unmarried | 1.927 | 1.042 | 3.562 | **0.036** | 1.921 | 0.989 | 3.731 | 0.054 |
| Current residence | Rural* |  |  |  |  |  |  |  |  |
|  | Urban | 0.440 | 0.237 | 0.817 | **0.009** | 0.481 | 0.252 | 0.919 | **0.027** |
| Monthly Income | More than 10,000 SAR | 0.799 | 0.332 | 1.920 | 0.616 | 0.752 | 0.293 | 1.931 | 0.554 |
|  | Less than 10,000 SAR | 0.444 | 0.258 | 0.764 | **0.003** | 0.483 | 0.272 | 0.859 | **0.013** |
|  | Nil* |  |  |  | **0.012** |  |  |  | **0.046** |
| Employment status | Healthcare workers | 0.806 | 0.348 | 1.865 | 0.614 | 0.894 | 0.370 | 2.163 | 0.804 |
|  | Students in healthcare profession | 1.236 | 0.555 | 2.752 | 0.604 | 1.400 | 0.611 | 3.209 | 0.427 |
|  | Non-healthcare workers/ unemployed* |  |  |  | 0.742 |  |  |  | 0.685 |

Supplementary table S7: Binary logistic regression for anxiety before and after AC19V with Sociodemographic variables as independent variables for the Saudi Arabian population. OR Odds ratio; 95% CI 95% confidence interval; AC19V advent of COVID-19 vaccine; * Reference category

| Variables | Sub-Categories | Before AC19V | | | | After Ac19V | | | |
| --- | --- | --- | --- | --- | --- | --- | --- | --- | --- |
|  |  | B value | 95% CI | | P Value | B value | 95% CI | | P Value |
|  |  |  | Lower | Upper |  |  | Lower | Upper |  |
| Age | - | 0.044 | -0.025 | 0.113 | 0.207 | 0.039 | -0.034 | 0.113 | 0.297 |
| Gender | Male* | - | - | - | - | - | - | - | - |
|  | Female | -0.502 | -1.851 | 0.846 | 0.465 | -0.464 | -1.906 | 0.977 | 0.528 |
| Educational status | Postgraduates and higher | 1.611 | -2.950 | 6.173 | 0.489 | 0.111 | -4.762 | 4.984 | 0.964 |
|  | Undergraduates | 0.451 | -3.349 | 4.252 | 0.816 | -0.540 | -4.600 | 3.520 | 0.794 |
|  | School level education* | -0.117 | -4.111 | 3.877 | 0.954 | -1.522 | -5.789 | 2.745 | 0.484 |
|  | Nil | - | - | - | - | - | - | - | - |
| Marital status | Married* | - | - | - | - | - | - | - | - |
|  | Unmarried | -0.574 | -1.992 | 0.845 | 0.428 | -0.462 | -1.979 | 1.055 | 0.550 |
| Current residence | Rural* |  |  |  |  |  |  |  |  |
|  | Urban | -0.809 | -2.499 | 0.881 | 0.348 | -0.865 | -2.671 | 0.941 | 0.348 |
| Monthly Income | More than 50,000 INR* | - | - | - | - | - | - | - | - |
|  | Less than 50,000 INR | -1.008 | -3.251 | 1.235 | 0.378 | -1.420 | -3.824 | 0.984 | 0.247 |
|  | Nil | -1.902 | -4.226 | 0.421 | 0.109 | -1.481 | -3.971 | 1.009 | 0.244 |
| Employment status | Healthcare workers | -1.129 | -3.151 | 0.894 | 0.274 | -0.782 | -2.944 | 1.381 | 0.479 |
|  | Students in healthcare profession | -0.679 | -2.782 | 1.425 | 0.527 | 0.852 | -1.397 | 3.102 | 0.458 |
|  | Non-healthcare workers/ unemployed* | - | - | - | - | - | - | - | - |

Supplementary table S8: Generalized linear regression analysis for PTSD before and after AC19V with Sociodemographic variables as independent variables for the Saudi Arabian population. 95% CI 95% confidence interval; AC19V advent of COVID-19 vaccine; * Reference category

| Variables | Sub-Categories | Before AC19V | | | | After Ac19V | | | |
| --- | --- | --- | --- | --- | --- | --- | --- | --- | --- |
|  |  | Crude OR | 95% CI | | P Value | Crude OR | 95% CI | | P Value |
|  |  |  | Lower | Upper |  |  | Lower | Upper |  |
| Age | - | 0.957 | 0.931 | 0.983 | **0.001** | 0.961 | 0.935 | 0.987 | **0.004** |
| Gender | Male* |  |  |  |  |  |  |  |  |
|  | Female | 1.691 | 1.045 | 2.738 | **0.032** | 1.842 | 1.129 | 3.003 | **0.014** |
| Educational status | Postgraduates and higher | 0.400 | 0.075 | 2.122 | 0.282 | 0.318 | 0.059 | 1.705 | 0.181 |
|  | Undergraduates | 0.432 | 0.105 | 1.770 | 0.243 | 0.386 | 0.094 | 1.584 | 0.186 |
|  | School level education | 0.409 | 0.093 | 1.790 | 0.235 | 0.382 | 0.087 | 1.675 | 0.202 |
|  | Nil* |  |  |  | 0.688 |  |  |  | 0.581 |
| Marital status | Married* |  |  |  |  |  |  |  |  |
|  | Unmarried | 1.636 | 0.985 | 2.719 | 0.057 | 1.618 | 0.969 | 2.703 | 0.066 |
| Current residence | Rural* |  |  |  |  |  |  |  |  |
|  | Urban | 0.419 | 0.227 | 0.775 | **0.006** | 0.491 | 0.269 | 0.895 | **0.020** |
| Monthly Income | More than 10,000 SAR | 0.539 | 0.233 | 1.245 | 0.148 | 0.556 | 0.237 | 1.302 | 0.176 |
|  | Less than 10,000 SAR | 0.682 | 0.421 | 1.106 | 0.121 | 0.778 | 0.480 | 1.261 | 0.308 |
|  | Nil* |  |  |  | 0.187 |  |  |  | 0.335 |
| Employment status | Healthcare workers | 1.196 | 0.588 | 2.435 | 0.621 | 1.035 | 0.505 | 2.120 | 0.925 |
|  | Students in healthcare profession | 2.111 | 0.987 | 4.514 | 0.054 | 2.017 | 0.952 | 4.274 | 0.067 |
|  | Non-healthcare workers/ unemployed* |  |  |  | 0.151 |  |  |  | 0.185 |

Supplementary table S9: Binary logistic regression for perceptive need for mental health support before and after AC19V with Sociodemographic variables as independent variables for the Saudi Arabian population. OR Odds ratio; 95% CI 95% confidence interval; AC19V advent of COVID-19 vaccine; * Reference category

| Variables | Sub-Categories | After AC19V | | | |
| --- | --- | --- | --- | --- | --- |
|  |  | Crude OR | 95% CI | | P Value |
|  |  |  | Lower | Upper |  |
| Age | - | 0.986 | 0.963 | 1.011 | 0.267 |
| Gender | Male* | - | - | - | - |
|  | Female | 1.650 | 1.025 | 2.656 | **0.039** |
| Educational status | Postgraduates and higher | 0.318 | 0.059 | 1.705 | 0.181 |
|  | Undergraduates | 0.612 | 0.149 | 2.511 | 0.496 |
|  | School level education | 0.804 | 0.183 | 3.535 | 0.773 |
|  | Nil* | - | - | - | 0.346 |
| Marital status | Married* | - | - | - | - |
|  | Unmarried | 1.033 | 0.626 | 1.702 | 0.900 |
| Current residence | Rural* | - | - | - | - |
|  | Urban | 0.593 | 0.319 | 1.103 | 0.099 |
| Monthly Income | More than 50,000 INR | 0.862 | 0.375 | 1.983 | 0.727 |
|  | Less than 50,000 INR | 0.631 | 0.387 | 1.031 | 0.066 |
|  | Nil* | - | - | - | 0.174 |
| Employment status | Healthcare workers | 1.125 | 0.546 | 2.319 | 0.750 |
|  | Students in healthcare profession | 0.513 | 0.242 | 1.087 | 0.082 |
|  | Non-healthcare workers/ unemployed* | - | - | - | 0.190 |

Supplementary table S10: Binary logistic regression for COVID-19 vaccine hesitancy with Sociodemographic variables as independent variables for the Saudi Arabian population. OR Odds ratio; 95% CI 95% confidence interval; AC19V advent of COVID-19 vaccine; * Reference category

| S. No | Variables | Crude OR (95% CI) | P value | Adjusted OR (95% CI) (for SD factors) | P value | Adjusted OR (95% CI) (for SD and COVID-19 related factors) | P value | Crude OR (95% CI) | P value | Adjusted OR (95% CI) (for SD factors) | P value | Adjusted OR (95% CI) (for SD and COVID-19 related factors) | P value |
| --- | --- | --- | --- | --- | --- | --- | --- | --- | --- | --- | --- | --- | --- |
| 1 | Tested positive for COVID 19 – YES  (Reference - No) | 1.258 (0.730-2.169) | 0.409 | 1.285 (0.727-2.272) | 0.388 | - | - | 1.332 (0.766-2.318) | 0.310 | 1.391 (0.756-2.558) | 0.289 | - | - |
| 2 | Taken COVID-19 vaccine  Yes-1^st^ dose | **0.374 (0.223-0.627)** | **<0.001** | **0.393 (0.232-0.666)** | **0.001** | - | - | **0.386 (0.168-0.887)** | **0.025** | 0.448 (0.189-1.064) | 0.069 | - | - |
|  | Yes -2^nd^ dose | **0.252 (0.134-0.475)** | **<0.001** | **0.291 (****0.149-0.565)** | **<0.001** | - | - | **0.445 (0.203-0.976)** | **0.043** | **0.372 (0.164-0.845)** | **0.018** | - | - |
|  | No (Reference) | - | **<0.001** | - | **<0.001** | - | - | - | 0.076 | - | 0.061 | - | - |
| 3 | Active infection after COVID-19 vaccine – yes | 0.487 (0.162-1.469) | 0.202 | 0.498 (0.162-1.536) | 0.225 | - | - | 0.438 (0.148-1.292) | 0.135 | 0.406 (0.128-1.290) | 0.126 | - | - |
|  | I don’t know | 0.693 (0.324-1.481) | 0.344 | 0.768 (0.351-1.682) | 0.510 | - | - | 0.430 (0.180-1.027) | 0.057 | 0.406 (0.164-1.003) | 0.051 | - | - |
|  | No | **0.283 (0.170-0.473)** | **<0.001** | **0.309 (0.182-0.522)** | **<0.001** | - | - | **0.419 (0.192-0.918)** | **0.030** | **0.397 (0.177-0.890)** | **0.025** | - | - |
|  | Not vaccinated (Reference) | - | **<0.001** | - | **<0.001** | - | - | - | 0.182 | - | 0.159 | - | - |
| 4 | Depression before | 1.035 (0.916-1.170) | 0.577 | 1.025 (0.902-1.165) | 0.702 | 1.029 (0.900-1.176) | 0.680 | **1.325 (1.164-1.508)** | **<0.001** | **1.358 (1.176-1.569)** | **<0.001** | **1.350 (1.167-1.563)** | **<0.001** |
| 5 | Anxiety before | 1.060 (0.946-1.188) | 0.318 | 1.062 (0.944-1.196) | 0.318 | 1.088 (0.962-1.230) | 0.179 | **1.368 (1.190-1.572)** | **<0.001** | **1.359 (1.168-1.582)** | **<0.001** | **1.344 (1.150-1.570)** | **<0.001** |
| 6 | PTSD before | 0.992 (0.959-1.026) | 0.651 | 0.988 (0.954-1.023) | 0.499 | 0.992 (0.956-1.028) | 0.654 | 1.017 (0.978-1.059) | 0.394 | 1.022 (.980-1.065) | 0.304 | 1.018 (0.976-1.062) | 0.407 |
| 7 | Depression after | 0.963 (0.862-1.075) | 0.497 | 0.945 (0.843-1.059) | 0.332 | 0.951 (0.845-1.071) | 0.406 | **1.201 (1.064-1.355)** | **0.003** | **1.209 (1.060-1.380)** | **0.005** | **1.200 (1.050-1.372)** | **0.008** |
| 8 | Anxiety after | 1.063 (0.952-1.187) | 0.275 | 1.048 (0.936-1.174) | 0.413 | 1.076 (0.956-1.211) | 0.223 | **1.454 (1.248-1.694)** | **<0.001** | **1.435 (1.221-1.687)** | **<0.001** | **1.409 (1.197-1.659)** | **<0.001** |
| 9 | PTSD after | 0.996 (0.966-1.027) | 0.785 | 0.988 (0.957-1.020) | 0.454 | 0.990 (0.958-1.024) | 0.569 | 1.030 (0.992-1.070) | 0.121 | 1.035 (0.996-1.076) | 0.083 | 1.033 (0.993-1.075) | 0.106 |
| 10 | Perceptive need for mental health support – before  Yes (reference- no) | 0.847 (0.562-1.276) | 0.427 | 0.865 (0.568-1.318) | 0.501 | 0.971 (0.625-1.507) | 0.894 | **2.120 (1.334-3.368)** | **0.001** | **2.069 (1.263-3.390)** | **0.004** | **2.053 (1.239-3.403)** | **0.005** |
| 11 | Perceptive need for mental health support – after  Yes (reference-no) | 1.054 (0.699-1.589) | 0.801 | 1.047 (0.687-1.596) | 0.831 | 1.184 (0.760-1.844) | 0.456 | **2.076 (1.303-3.309)** | **0.002** | **2.054 (1.252-3.370)** | **0.004** | **1.958 (1.184-3.238)** | **0.009** |

Supplementary Table S11: Binary logistic regression analysis of COVID-19 vaccine hesitancy (regression models 1, 2, and 3). Significant P values are mentioned in bold (P< 0.05). Regression model 1 - Crude odds ratio (95% CI) and P value, Regression model 2 - Adjusted OR (95% CI) (for SD factors), Regression model 3 - Adjusted OR (95% CI) (for SD and COVID-19 related factors). Gray shaded portions indicate results of Saudi data and unshaded portions indicate results of Indian data. OR Odds ratio; 95% CI 95% confidence interval; SD factors Sociodemographic factors; AC19V advent of COVID-19 vaccine; PTSD Post traumatic stress disorder

| S. No | Variables | Crude OR (95% CI) | P value | Adjusted OR (95% CI) (for SD factors) | P value | Adjusted OR (95% CI) (for SD and COVID related factors) | P value | Crude OR (95% CI) | P value | Adjusted OR (95% CI) (for SD factors) | P value | Adjusted OR (95% CI) (for SD and COVID related factors) | P value |
| --- | --- | --- | --- | --- | --- | --- | --- | --- | --- | --- | --- | --- | --- |
| 1 | Tested positive for COVID 19 – YES  (Reference - No) | 0.659 (0.361-1.205) | 0.176 | 0.705 (0.379-1.312) | 0.271 | - | - | 0.877 (0.482-1.595) | 0.668 | 1.004 (0.507-1.988) | 0.991 | - | - |
| 2 | Taken COVID-19 vaccine  Yes-1^st^ dose | 1.373 (0.818-2.304) | 0.230 | 1.403 (0.824-2.389) | 0.213 | - | - | 0.688 (0.313-1.510) | 0.351 | 0.642 (0.271-1.519) | 0.313 | - | - |
|  | Yes -2^nd^ dose | 0.934 (0.488-1.789) | 0.837 | 1.026 (0.515-2.045) | 0.941 | - | - | 0.559 (0.268-1.166) | 0.121 | 0.473 (0.212-1.055) | 0.067 | - | - |
|  | No (Reference) | - | 0.284 | - | 0.357 | - | - | - | 0.285 | - | 0.175 | - | - |
| 3 | Active infection after COVID-19 vaccine – yes | 1.489 (0.486-4.559) | 0.486 | 1.398 (0.449-4.351) | 0.563 | - | - | 0.386 (0.118-1.261) | 0.115 | 0.293 (0.078-1.095) | 0.068 | - | - |
|  | I don’t know | 1.374 (0.645-2.930) | 0.410 | 1.465 (0.670-3.201) | 0.339 | - | - | 0.624 (0.270-1.445) | 0.271 | 0.609 (0.243-1.524) | 0.289 | - | - |
|  | No | 1.195 (0.716-1.996) | 0.495 | 1.263 (0.743-2.147) | 0.389 | - | - | 0.627 (0.302-1.302) | 0.210 | 0.539 (0.246-1.178) | 0.121 | - | - |
|  | Not vaccinated (Reference) | - | 0.802 | - | 0.753 | - | - | - | 0.423 | - | 0.264 | - | - |
| 4 | COVID-19 vaccine hesitancy | 0.981 (0.942-1.021) | 0.349 | 0.978 (0.938-1.020) | 0.305 | 0.979 (0.937-1.022) | 0.329 | **1.038 (1.008-1.068)** | **0.012** | **1.037 (1.006-1.069)** | **0.020** | **1.033 (1.001-1.067)** | **0.041** |
| 5 | Depression before AC19V | **2.768 (2.236-3.427)** | **<0.001** | **2.905 (2.325-3.630)** | **<0.001** | **2.929 (2.337-3.670)** | **<0.001** | **2.270 (1.878-2.745)** | **<0.001** | **2.250 (1.840-2.751)** | **<0.001** | **2.280 (1.856-2.800)** | **<0.001** |
| 6 | Anxiety before AC19V | **1.871 (1.610-2.174)** | **<0.001** | **1.881 (1.611-2.197)** | **<0.001** | **1.877 (1.607-2.192)** | **<0.001** | **1.894 (1.607-2.231)** | **<0.001** | **1.971 (1.637-2.372)** | **<0.001** | **1.997 (1.649-2.419)** | **<0.001** |
| 7 | PTSD before AC19V | **1.120 (1.076-1.167)** | **<0.001** | **1.125 (1.079-1.173)** | **<0.001** | **1.126 (1.079-1.175)** | **<0.001** | **1.059 (1.014-1.106)** | **0.009** | **1.073 (1.023-1.124)** | **0.003** | **1.068 (1.019-1.120)** | **0.006** |
| 8 | Anxiety after AC19V | **2.202 (1.861-2.606)** | **<0.001** | **2.204 (1.857-2.616)** | **<0.001** | **2.204 (1.853-2.621)** | **<0.001** | **2.455 (1.981-3.044)** | **<0.001** | **2.709 (2.103-3.490)** | **<0.001** | **2.764 (2.128-3.591)** | **<0.001** |
| 9 | PTSD after AC19V | **1.136 (1.093-1.181)** | **<0.001** | **1.142 (1.097-1.188)** | **<0.001** | **1.142 (1.097-1.189)** | **<0.001** | **1.084 (1.041-1.130)** | **<0.001** | **1.090 (1.042-1.140)** | **<0.001** | **1.088 (1.040-1.138)** | **<0.001** |
| 10 | Perceptive need for mental health support – before AC19V  Yes (reference- no) | **3.026 (1.943-4.712)** | **<0.001** | **3.064 (1.949-4.818)** | **<0.001** | **3.125 (1.969-4.958)** | **<0.001** | **5.072 (2.961-8.691)** | **<0.001** | **5.117 (2.860-9.154)** | **<0.001** | **5.048 (2.790-9.133)** | **<0.001** |
| 11 | Perceptive need for mental health support – after AC19V  Yes (reference-no) | **3.925 (2.499-6.167)** | **<0.001** | **3.913 (2.468-6.206)** | **<0.001** | **3.970 (2.476-6.365)** | **<0.001** | **3.889 (2.320-6.518)** | **<0.001** | **3.758 (2.155-6.553)** | **<0.001** | **3.607 (2.052-6.341)** | **<0.001** |

Supplementary Table S12: Binary logistic regression analysis of depression after AC19V (regression models 1, 2, and 3). Significant P values are mentioned in bold (P< 0.05). Regression model 1 - Crude odds ratio (95% CI) and P value, Regression model 2 - Adjusted OR (95% CI) (for SD factors), Regression model 3 - Adjusted OR (95% CI) (for SD and COVID-19 related factors). Gray shaded portions indicate results of Saudi data and unshaded portions indicate results of Indian data. OR Odds ratio; 95% CI 95% confidence interval; SD factors Sociodemographic factors; AC19V advent of COVID-19 vaccine; PTSD Post traumatic stress disorder

| S. No | Variables | Crude OR (95% CI) | P value | Adjusted OR (95% CI) (for SD factors) | P value | Adjusted OR (95% CI) (for SD and COVID related factors) | P value | Crude OR (95% CI) | P value | Adjusted OR (95% CI) (for SD factors) | P value | Adjusted OR (95% CI) (for SD and COVID related factors) | P value |
| --- | --- | --- | --- | --- | --- | --- | --- | --- | --- | --- | --- | --- | --- |
| 1 | Tested positive for COVID 19 – YES  (Reference - No) | **0.419 (0.198- 0.884)** | **0.022** | **0.438 (0.204-0.943)** | **0.035** | - | - | 1.247 (0.663-2.344) | 0.494 | 1.705 (0.813-3.576) | 0.158 | - | - |
| 2 | Taken COVID-19 vaccine  Yes-1^st^ dose | 1.567 (0.877-2.801) | 0.129 | 1.714 (0.944-3.115) | 0.077 | - | - | **0.235 (0.098-0.567)** | **0.001** | **0.247 (0.096-0.635)** | **0.004** | - | - |
|  | Yes -2^nd^ dose | 1.328 (0.654- 2.693) | 0.432 | 1.712 (0.810-3.615) | 0.159 | - | - | **0.438 (0.208-0.922)** | **0.030** | **0.370 (0.163-0.840)** | **0.018** | - | - |
|  | No (Reference) | - | 0.313 | - | 0.187 | - | - | - | **0.006** | - | **0.011** | - | - |
| 3 | Active infection after COVID-19 vaccine – yes | 1.400 (0.403- 4.865) | 0.597 | 1.390 (0.390-4.949) | 0.612 | - | - | **0.210 (0.053-0.830)** | **0.026** | **0.191 (0.041-0.884)** | **0.034** | - | - |
|  | I don’t know | 1.925 (0.858- 4.320) | 0.112 | 2.283 (0.986-5.287) | 0.054 | - | - | 0.460 (0.194-1.091) | 0.078 | 0.455 (0.175-1.180) | 0.105 | - | - |
|  | No | 1.429 (0.803- 2.540) | 0.224 | 1.651 (0.910-2.993) | 0.099 | - | - | **0.349 (0.164-0.742)** | **0.006** | **0.304 (0.134-0.688)** | **0.004** | - | - |
|  | Not vaccinated (Reference) | - | 0.435 | - | 0.234 | - | - | - | **0.030** | - | **0.022** | - | - |
| 4 | VHS12 | **1.054 (1.007- 1.103)** | **0.025** | **1.048 (1.000-1.099)** | **0.049** | **1.058 (1.007-1.111)** | **0.024** | **1.051 (1.019-1.085)** | **0.002** | **1.046 (1.011-1.081)** | **0.009** | **1.037 (1.002-1.074)** | **0.038** |
| 5 | PHQ2 before | **2.050 (1.717-** **2.449)** | **<0.001** | **2.142 (1.776-2.583)** | **<0.001** | **2.210 (1.818-2.685)** | **<0.001** | **2.026 (1.689-2.431)** | **<0.001** | **2.003 (1.644-2.441)** | **<0.001** | **2.069 (1.677-2.553)** | **<0.001** |
| 6 | GAD2 before | **2.651 (2.164-** **3.248)** | **<0.001** | **2.932 (2.332-3.686)** | **<0.001** | **2.993 (2.362-3.792)** | **<0.001** | **2.662 (2.128-3.330)** | **<0.001** | **2.630 (2.077-3.331)** | **<0.001** | **2.676 (2.092-3.424)** | **<0.001** |
| 7 | IES6 before | **1.141 (1.090-1.194)** | **<0.001** | **1.148 (1.095-1.203)** | **<0.001** | **1.149 (1.095-1.205)** | **<0.001** | **1.113 (1.059-1.170)** | **<0.001** | **1.134 (1.074-1.197)** | **<0.001** | **1.134 (1.073-1.200)** | **<0.001** |
| 8 | PHQ2 after | **2.270 (1.900-2.713)** | **<0.001** | **2.303 (1.917-2.766)** | **<0.001** | **2.348 (1.943-2.838)** | **<0.001** | **2.614 (2.103-3.251)** | **<0.001** | **2.775 (2.165-3.556)** | **<0.001** | **2.900 (2.229-3.773)** | **<0.001** |
| 9 | IES6 after | **1.149 (1.102-1.199)** | **<0.001** | **1.153 (1.104-1.204)** | **<0.001** | **1.152 (1.102-1.204)** | **<0.001** | **1.118 (1.067-1.171)** | **<0.001** | **1.130 (1.075-1.188)** | **<0.001** | **1.131 (1.074-1.191)** | **<0.001** |
| 10 | Perceptive need for mental health support – before  Yes (reference- no) | **2.965 (1.836-4.790)** | **<0.001** | **3.079 (1.881-5.039)** | **<0.001** | **2.991 (1.811-4.942)** | **<0.001** | **3.993 (2.213-7.207)** | **<0.001** | **3.431 (1.844-6.384)** | **<0.001** | **3.669 (1.919-7.016)** | **<0.001** |
| 11 | Perceptive need for mental health support – after  Yes (reference-no) | **2.965 (1.836-4.790)** | **<0.001** | **2.978 (1.822-4.870)** | **<0.001** | **2.961 (1.789-4.902)** | **<0.001** | **3.540 (1.997-6.275)** | **<0.001** | **3.016 (1.654-5.503)** | **<0.001** | **2.983 (1.597-5.572)** | **<0.001** |

Supplementary Table S13: Binary logistic regression analysis of anxiety after AC19V (regression models 1, 2, and 3). Significant P values are mentioned in bold (P< 0.05). Regression model 1 - Crude odds ratio (95% CI) and P value, Regression model 2 - Adjusted OR (95% CI) (for SD factors), Regression model 3 - Adjusted OR (95% CI) (for SD and COVID-19 related factors). Gray shaded portions indicate results of Saudi data and unshaded portions indicate results of Indian data. OR Odds ratio; 95% CI 95% confidence interval; SD factors Sociodemographic factors; AC19V advent of COVID-19 vaccine; PTSD Post traumatic stress disorder

| S. No | Variables | Crude B (95% Wald CI) | P value | Adjusted B (95% Wald CI) (for SD factors) | P value | Adjusted B (95% Wald CI) (for SD and COVID related factors) | P value | Crude B (95% Wald CI) | P value | Adjusted B (95% Wald CI) (for SD factors) | P value | Adjusted B (95% Wald CI) (for SD and COVID related factors) | P value |
| --- | --- | --- | --- | --- | --- | --- | --- | --- | --- | --- | --- | --- | --- |
| 1 | Tested positive for COVID 19 – YES  (Reference - No) | -0.990 (-2.782 to 0.802) | 0.279 | -1.299 (-3.100 to 0.502) | 0.157 | - | - | -0.192 (-1.848 to 1.463) | 0.820 | -0.527 (-2.295 to 1.240) | 0.559 | - | - |
| 2 | Taken COVID-19 vaccine  Yes-1^st^ dose | 0.517 (-1.092 to 2.126) | 0.529 | 0.795 (-0.811 to 2.401) | 0.332 | - | - | -1.062 (-3.380 to 1.257) | 0.370 | -1.166 (-3.515 to 1.183) | 0.331 | - | - |
|  | Yes -2^nd^ dose | -0.068 (-2.041 to 1.906) | 0.946 | 0.401 (-1.632 to 2.434) | 0.699 | - | - | -1.143 (-3.306 to 1.020) | 0.300 | -1.231 (-3.417 to 0.955) | 0.270 | - | - |
|  | No (Reference) | - | - | - | - | - | - | - | - | - | - | - | - |
| 3 | Active infection after COVID-19 vaccine – yes | 0.278 (-3.324 to 3.880) | 0.880 | 0.180 (-3.389 to 3.748) | 0.921 | - | - | -2.231 (-5.353 to 0.891) | 0.161 | -2.685 (-5.899 to 0.529) | 0.102 | - | - |
|  | I don’t know | 0.493 (-1.905 to 2.891) | 0.687 | 0.877 (-1.522 to 3.275) | 0.474 | - | - | -0.368 (-2.808 to 2.071) | 0.767 | -0.403 (-2.859 to 2.054) | 0.748 | - | - |
|  | No | 0.329 (-1.256 to 1.913) | 0.684 | 0.699 (-0.890 to 2.288) | 0.389 | - | - | -1.246 (-3.399 to .907) | 0.257 | -1.314 (-3.461 to 0.833) | 0.230 | - | - |
|  | Not vaccinated (Reference) | - | - | - | - | - | - | - | - | - | - | - | - |
| 4 | VHS12 | -0.022 (-0.150 to 0.106) | 0.732 | -0.050 (-0.177 to 0.078) | 0.445 | -0.044 (-0.174 to 0.087) | 0.510 | 0.028 (-0.052 to 0.108) | 0.499 | 0.030 (-0.051 to 0.111) | 0.469 | 0.023 (-0.059 to 0.106) | 0.580 |
| 5 | PHQ2 before | **1.116 (0.727 to 1.505)** | **<0.001** | **1.131 (0.741 to 1.521)** | **<0.001** | **1.127 (0.735 to 1.520)** | **<0.001** | **0.470 (0.116 to 0.824)** | **0.009** | **0.547 (0.171 to 0.922)** | **0.004** | **0.544 (0.168 to 0.921)** | **0.005** |
| 6 | GAD2 before | **1.329 (0.978 to 1.680)** | **<0.001** | **1.374 (1.026 to 1.722)** | **<0.001** | **1.362 (1.014 to 1.711)** | **<0.001** | **1.005 (0.654 to 1.356)** | **<0.001** | **1.180 (0.809 to 1.552)** | **<0.001** | **1.193 (0.814 to 1.571)** | **<0.001** |
| 7 | IES6 before | **0.923 (0.862 to 0.985)** | **<0.001** | **0.914 (0.853 to 0.975)** | **<0.001** | **0.917 (0.857 to 0.978)** | **<0.001** | **0.791 (0.711 to 0.872)** | **<0.001** | **0.795 (0.714 to 0.876)** | **<0.001** | **0.796 (0.716 to 0.877)** | **<0.001** |
| 8 | PHQ2 after | **1.515 (1.185 to 1.845)** | **<0.001** | **1.511 (1.182 to 1.840)** | **<0.001** | **1.504 (1.174 to 1.835)** | **<0.001** | **0.843 (0.507 to 1.178)** | **<0.001** | **0.920 (0.572 to 1.268)** | **<0.001** | **0.905 (0.557 to 1.254)** | **<0.001** |
| 9 | GAD2 after | **1.635 (1.313 to 1.958)** | **<0.001** | **1.638 (1.319 to 1.958)** | **<0.001** | **1.628 (1.306 to 1.949)** | **<0.001** | **1.273 (.920 to 1.627)** | **<0.001** | **1.427 (1.062 to 1.791)** | **<0.001** | **1.432 (1.062 to 1.801)** | **<0.001** |
| 10 | Perceptive need for mental health support – before  Yes (reference- no) | **3.231 (1.912 to 4.549)** | **<0.001** | **3.468 (2.173 to 4.763)** | **<0.001** | **3.447 (2.140 to 4.755)** | **<0.001** | **1.492 (0.128 to 2.855)** | **0.032** | **1.584 (0.180 to 2.988)** | **0.027** | **1.514 (0.095 to 2.934)** | **0.037** |
| 11 | Perceptive need for mental health support – after  Yes (reference-no) | **3.351 (2.036 to 4.667)** | **<0.001** | **3.503 (2.205 to 4.800)** | **<0.001** | **3.539 (2.224 to 4.853)** | **<0.001** | **1.516 (.145 to 2.886)** | **0.030** | **1.605 (0.202 to 3.008)** | **0.025** | **1.534 (0.121 to 2.946)** | **0.033** |

Supplementary Table S14: Generalized linear regression analysis of PTSD after AC19V (regression models 1, 2, and 3). Significant P values are mentioned in bold (P< 0.05). Regression model 1 - Crude odds ratio (95% CI) and P value, Regression model 2 - Adjusted OR (95% CI) (for SD factors), Regression model 3 - Adjusted OR (95% CI) (for SD and COVID-19 related factors). Gray shaded portions indicate results of Saudi data and unshaded portions indicate results of Indian data. OR Odds ratio; 95% CI 95% confidence interval; SD factors Sociodemographic factors; AC19V advent of COVID-19 vaccine; PTSD Post traumatic stress disorder

| S. No | Variables | Crude OR (95% CI) | P value | Adjusted OR (95% CI) (for SD factors) | P value | Adjusted OR (95% CI) (for SD and COVID related factors) | P value | Crude OR (95% CI) | P value | Adjusted OR (95% CI) (for SD factors) | P value | Adjusted OR (95% CI) (for SD and COVID related factors) | P value |
| --- | --- | --- | --- | --- | --- | --- | --- | --- | --- | --- | --- | --- | --- |
| 1 | Tested positive for COVID 19 – YES  (Reference - No) | 0.979 (0.567- 1.693) | 0.941 | 1.067 (0.604 to 1.883) | 0.824 | - | - | 0.826 (0.476-1.434) | 0.498 | 1.016 (0.550-1.878) | 0.960 | - | - |
| 2 | Taken COVID-19 vaccine  Yes-1^st^ dose | **2.229 (1.329- 3.737)** | **0.002** | **2.277 (1.339-3.870)** | **0.002** | - | - | 0.575 (0.266-1.244) | 0.160 | 0.664 (0.295-1.495) | 0.323 | - | - |
|  | Yes -2^nd^ dose | **2.010 (1.079-3.744)** | **0.028** | **2.098 (1.084-4.061)** | **0.028** | - | - | **0.448 (0.217-0.923)** | **0.030** | **0.417 (0.194-0.897)** | **0.025** | - | - |
|  | No (Reference) | - | **0.009** | - | **0.008** | - | - | - | 0.085 | - | 0.052 | - | - |
| 3 | Active infection after COVID-19 vaccine – yes | **4.690 (1.473- 14.933)** | **0.009** | **4.677 (1.446-15.134)** | **0.010** | - | - | **0.281 (0.094-0.842)** | **0.023** | **0.278 (0.084-0.918)** | **0.036** | - | - |
|  | I don’t know | **2.345 (1.113- 4.939)** | **0.025** | **2.508 (1.158-5.430)** | **0.020** | - | - | **0.406 (0.178-0.923)** | **0.032** | 0.423 (0.179-1.003) | 0.051 | - | - |
|  | No | **2.024 (1.216- 3.370)** | **0.007** | **2.059 (1.217-3.483)** | **0.007** | - | - | 0.562 (0.274-1.155) | 0.117 | 0.560 (0.266-1.182) | 0.128 | - | - |
|  | Not vaccinated (Reference) | - | **0.009** | - | **0.009** | - | - | - | 0.077 | - | 0.128 | - | - |
| 4 | VHS12 | 1.020 (0.981- 1.061) | 0.325 | 1.020 (0.980-1.062) | 0.325 | 1.031 (0.988-1.075) | 0.159 | **1.046 (1.017-1.076)** | **0.002** | **1.047 (1.017-1.078)** | **0.002** | **1.043 (1.012-1.075)** | **0.006** |
| 5 | PHQ2 before | **1.408 (1.232-** **1.608)** | **<0.001** | **1.419 (1.236-1.628)** | **<0.001** | **1.420 (1.234-1.634)** | **<0.001** | **1.693 (1.462-1.961)** | **<0.001** | **1.683 (1.436-1.972)** | **<0.001** | **1.664 (1.418-1.953)** | **<0.001** |
| 6 | GAD2 before | **1.620 (1.411-** **1.859)** | **<0.001** | **1.611 (1.400-1.853)** | **<0.001** | **1.628 (1.411-1.878)** | **<0.001** | **1.645 (1.416-1.912)** | **<0.001** | **1.624 (1.380-1.911)** | **<0.001** | **1.654 (1.394-1.964)** | **<0.001** |
| 7 | IES6 before | **1.070 (1.032-1.109)** | **<0.001** | **1.075 (1.036-1.116)** | **<0.001** | **1.079 (1.039-1.121)** | **<0.001** | 1.040 (1.000-1.083) | 0.053 | **1.048 (1.005-1.094)** | **0.029** | 1.043 (0.999-1.089) | 0.054 |
| 8 | PHQ2 after | **1.568 (1.377-1.785)** | **<0.001** | **1.579 (1.382-1.804)** | **<0.001** | **1.583 (1.382-1.812)** | **<0.001** | **1.613 (1.402-1.854)** | **<0.001** | **1.600 (1.378-1.858)** | **<0.001** | **1.598 (1.371-1.862)** | **<0.001** |
| 9 | GAD2 after | **1.551 (1.362-1.765)** | **<0.001** | **1.558 (1.365-1.779)** | **<0.001** | **1.564 (1.367-1.790)** | **<0.001** | **1.613 (1.382-1.882)** | **<0.001** | **1.569 (1.335-1.845)** | **<0.001** | **1.564 (1.325-1.846)** | **<0.001** |
| 10 | IES6 after | **1.083 (1.048-1.120)** | **<0.001** | **1.091 (1.054-1.130)** | **<0.001** | **1.093 (1.055-1.132)** | **<0.001** | **1.042 (1.003-1.082)** | **0.033** | **1.045 (1.005-1.087)** | **0.028** | **1.044 (1.003-1.087)** | **0.034** |

Supplementary Table S15: Binary logistic regression analysis of perceptive need for mental health support (regression models 1, 2, and 3). Significant P values are mentioned in bold (P< 0.05). Regression model 1 - Crude odds ratio (95% CI) and P value, Regression model 2 - Adjusted OR (95% CI) (for SD factors), Regression model 3 - Adjusted OR (95% CI) (for SD and COVID-19 related factors). Gray shaded portions indicate results of Saudi data and unshaded portions indicate results of Indian data. OR Odds ratio; 95% CI 95% confidence interval; SD factors Sociodemographic factors; AC19V advent of COVID-19 vaccine; PTSD Post traumatic stress disorder

| **Rotated Component Matrix^a^** | | |
| --- | --- | --- |
|  | Component | |
|  | 1 | 2 |
| 1. COVID-19 Vaccines are important for my health | .741 |  |
| 2. COVID-19 Vaccines are effective | .708 |  |
| 3. Being vaccinated against COVID-19 is important for the health of others in my community | .840 |  |
| 4. All COVID-19 vaccines offered by the government programme/campaign in my community are beneficial | .695 |  |
| 5. Newly introduced COVID-19 vaccines carry more risks than older vaccines |  | .676 |
| 6. The information I receive about COVID-19 vaccines from the vaccine programme is reliable and trustworthy | .631 |  |
| 7. Getting COVID-19 vaccines is a good way to protect myself from disease | .793 |  |
| 8. Generally, I do what my doctor or health care provider recommends about vaccines | .692 |  |
| 9. I am concerned about serious adverse effects of COVID-19 vaccines |  | .709 |
| 10. I am uncomfortable getting a COVID-19 vaccine that was rushed into production |  | .630 |
| 11. Corporations manufacturing COVID-19 vaccines only care for profit |  | .691 |
| 12. COVID-19 Vaccines cause diseases |  | .585 |
| Extraction Method: Principal Component Analysis.  Rotation Method: Varimax with Kaiser Normalization. | | |
| a. Rotation converged in 3 iterations. | | |

Supplementary table 16: Principal Component Analysis of COVID19-VHS12 scale of English version

| **Rotated Component Matrix^a^** | | |
| --- | --- | --- |
|  | Component | |
|  | 1 | 2 |
| 1. COVID-19 Vaccines are important for my health | .825 |  |
| 2. COVID-19 Vaccines are effective | .785 |  |
| 3. Being vaccinated against COVID-19 is important for the health of others in my community | .889 |  |
| 4. All COVID-19 vaccines offered by the government programme/campaign in my community are beneficial | .833 |  |
| 5. Newly introduced COVID-19 vaccines carry more risks than older vaccines |  | .721 |
| 6. The information I receive about COVID-19 vaccines from the vaccine programme is reliable and trustworthy | .822 |  |
| 7. Getting COVID-19 vaccines is a good way to protect myself from disease | .863 |  |
| 8. Generally, I do what my doctor or health care provider recommends about vaccines | .828 |  |
| 9. I am concerned about serious adverse effects of COVID-19 vaccines |  | .782 |
| 10. I am uncomfortable getting a COVID-19 vaccine that was rushed into production |  | .741 |
| 11. Corporations manufacturing COVID-19 vaccines only care for profit |  | .676 |
| 12. COVID-19 Vaccines cause diseases |  | .613 |
| Extraction Method: Principal Component Analysis.  Rotation Method: Varimax with Kaiser Normalization. | | |
| a. Rotation converged in 3 iterations. | | |

Supplementary table 17: Principal Component Analysis of COVID19-VHS12 scale of Arabic version

## Supplementary Figures


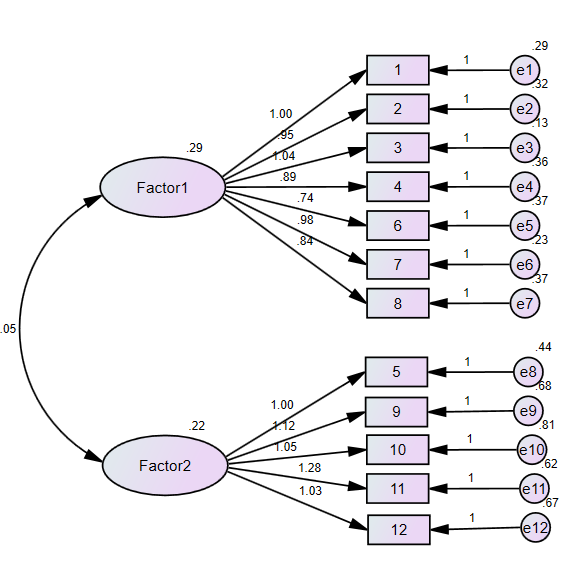


**Supplementary Figure S1.** Confirmatory factor analysis of two factors obtained from EFA of English version of COVID19-VHS12


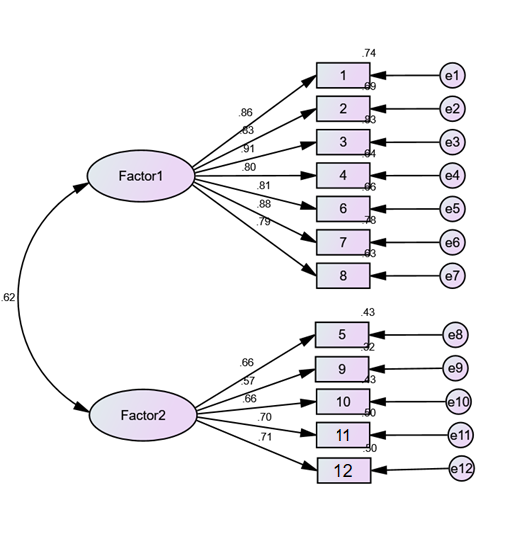


**Supplementary Figure S2.** Confirmatory factor analysis of two factors obtained from EFA of Arabic version of COVID19-VHS12


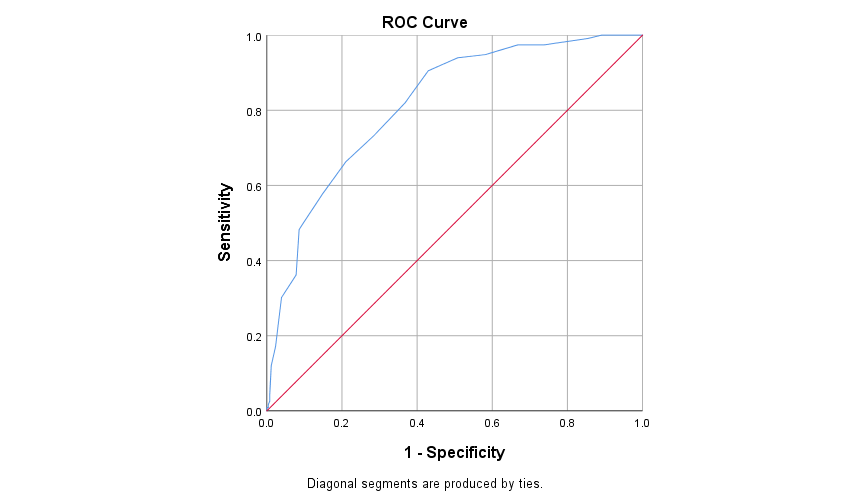


**Supplementary Figure S3.** Receiver operator Curve of COVID19-VHS12 Scale of English version


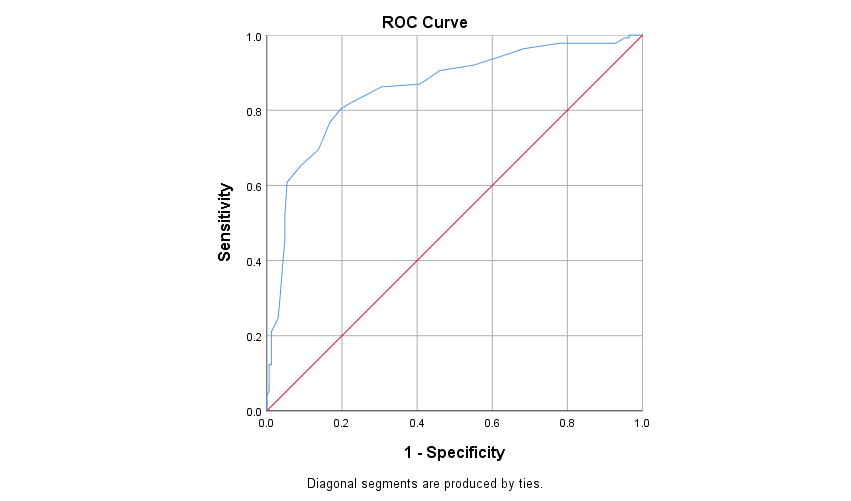


**Supplementary Figure S4.** Receiver operator Curve of COVID19-VHS12 Scale of Arabic version
